# A cryo-EM processing pipeline for microtubules using *CryoSPARC*

**DOI:** 10.1107/S2059798326003062

**Published:** 2026-04-27

**Authors:** Daniel Zhang, Hugo Muñoz-Hernández, Pavel Filipcik, Kushal Sejwal, Yixin Xu, Sung Ryul Choi, Michel O. Steinmetz, Michal Wieczorek

**Affiliations:** a Institute of Molecular Biology and Biophysics ETH Zürich Zürich Switzerland; b PSI Center for Life Sciences Paul Scherrer Institut Villigen Switzerland; c Biozentrum University of Basel 4056Basel Switzerland; Princeton University, USA

**Keywords:** cryo-EM, microtubules, helical reconstructions, tubulin, single-particle analysis

## Abstract

Microtubules are cytoskeletal filaments that are typically characterized by a discontinuous helical lattice of α/β-tubulin heterodimers. Microtubules can also adopt variable lattice architectures both *in vitro* and in cellular contexts. Pseudo-helical averaging processing strategies have been developed to generate cryo-EM reconstructions of microtubules with and without decorating protein-binding partners, but these pipelines can be difficult to implement for the average user, especially for undecorated filaments. Here, we describe *MiCSPARC*, a cryo-EM processing pipeline developed around *CryoSPARC* [Punjani *et al.* (2017 ▸), *Nat. Methods*, **14**, 290–296], which leverages automated particle picking and fast 3D refinement times in *CryoSPARC* to determine the structures of both decorated and undecorated microtubules. We generate reconstructions of undecorated GDP microtubules, as well as kinesin-1 motor domain-decorated GMPCPP filaments, at resolutions of up to 2.8 Å, demonstrating the robustness of the pipeline. Based on its convenient implementation and its ability to routinely generate high-resolution, seam-corrected microtubule reconstructions, *MiCSPARC* should provide a valuable tool for understanding microtubule dynamics, microtubule-associated proteins and microtubule-targeting agents.

## Abstract 2

Cryo-EM reconstructions of microtubules are currently technically challenging and time-consuming for average users. A cryo-EM processing pipeline for microtubules using *CryoSPARC* has been developed that overcomes many of these current issues.

## 1. Introduction

Microtubules are cytoskeletal polymers built from α/β-tubulin heterodimers, which self-associate in a head-to-tail manner into linear, polarized protofilaments; β-tubulin, a site of GTP binding and hydrolysis, points towards the fast-polymerizing or ‘plus’ end of the microtubule, while α-tubulin, containing a non-exchangeable GTP-binding site, points towards the slow-polymerizing or ‘minus’ end (reviewed in Brouhard & Rice, 2014 ▸; Akhmanova & Steinmetz, 2015 ▸). In canonical microtubules, 13 α/β-tubulin protofilaments associate laterally in the B-type configuration, resulting in a hollow cylindrical structure and a ‘seam’ that forms between only two protofilaments marked by heterotypic A-type α- and β-tubulin lateral contacts (Wade *et al.*, 1990 ▸; Sui & Downing, 2010 ▸). The seam therefore complicates helical averaging in cryo-electron microscopy (cryo-EM) single-particle analysis (SPA) because the α- and β-tubulin monomers are structurally similar and are difficult to distinguish from one another during classifications. Introducing a decorating protein that forms a 1:1 complex with each α/β-tubulin heterodimer can help to identify the seam (Zhang & Nogales, 2015 ▸), but can also physically distort the microtubule lattice (Lacey *et al.*, 2019 ▸; Zhang *et al.*, 2018 ▸). Moreover, differences in microtubule lattice architectures, including, for example, variable protofilament numbers, changes in tubulin dimer spacing along the lattice and supertwists along the filament, as well as partial binding occupancy of associated proteins to *in vitro* polymerized microtubules, all further limit achievable resolutions and the interpretability of reconstructions generated with conventional processing pipelines.

Several strategies have been developed to determine the structures of decorated microtubules (Cook *et al.*, 2020 ▸; Zhang & Nogales, 2015 ▸; Debs *et al.*, 2020 ▸). However, these methods experience limitations that make routine and fast microtubule reconstructions difficult to achieve for average cryo-EM users. For example, accurate 3D references containing correctly positioned decorators relative to α/β-tubulin are crucial for initial alignments, but current methods use either existing structures as references based on loose structural similarity (Cook *et al.*, 2020 ▸), or else generate references entirely *in silico* (Zhang & Nogales, 2015 ▸), both of which can introduce potential sources of bias. Particle pre-averaging is also used to increase the signal to noise for initial alignments (Cook *et al.*, 2020 ▸; Zhang & Nogales, 2015 ▸), but adds unnecessary complexity, since the so-called ‘super-particles’ need to first be well aligned in 2D to start being truly helpful in subsequent processing steps. Lastly, existing strategies do not yet take advantage of the accessibility and previously noted faster refinement times in software packages such as *CryoSPARC* (Punjani *et al.*, 2017 ▸). As a result, it remains difficult and time-consuming to determine cryo-EM structures of decorated microtubules, and seam-corrected, undecorated filament reconstructions remain out of reach for most users, limiting potential progress in the field of microtubule structural biology.

Here, we describe *MiCSPARC*, a microtubule processing pipeline developed around *CryoSPARC* that leverages improved automated particle picking, protofilament number sorting, seam searching and the faster 3D refinement times in *CryoSPARC* to accurately reconstruct both decorated and undecorated microtubules. We provide example *MiCSPARC* reconstructions of seam-corrected microtubules, including a seam-corrected, undecorated filament at an overall ∼3.6 Å resolution, with α/β-tubulin in a single protofilament reconstruction reaching 3.0 Å. The ability of *MiCSPARC* to reliably generate high-quality microtubule reconstructions should accelerate structural studies of undecorated microtubules and filaments in complex with microtubule-associated proteins or microtubule-targeting agents.

## 2. Methods

### 2.1. Protein purification

Calf brain tubulin used in the KIF5A motor domain-decorated dataset was purchased from the Centro de Investigaciones Biológicas Margarita Salas (Microtubule Stabilizing Agent Group), CSIC, Madrid, Spain. Porcine brain tubulin used in the undecorated dataset was purified from brain tissue following the method of Castoldi & Popov (2003 ▸).

Human kinesin KIF5A motor domain (residues 1–300; insert prepared from cDNA kindly provided by C. Hoogenraad) in PSTCm1 vector (Olieric *et al.*, 2010 ▸) was expressed in *Escherichia coli* Rosetta2 (Novagen) cells grown at 37°C in LB medium supplemented with 50 µg ml^−1^ kanamycin and 30 µg ml^−1^ chloramphenicol to an OD_600_ of 0.4–0.6. Expression was induced with 0.4 m*M* isopropyl β-d-1-thiogalactopyranoside (IPTG; Sigma–Aldrich) and the cultures were incubated overnight at 20°C. The cell pellets were resuspended in lysis buffer [50 m*M* HEPES pH 8.0, 500 m*M* NaCl, 10 m*M* imidazole, 10% glycerol, 2 m*M* β-mercaptoethanol and one cOmplete EDTA-free protease-inhibitor cocktail tablet (Roche)] and lysed on ice by ultrasonication. The lysates were clarified by ultracentrifugation, and the resulting supernatants were filtered through a 0.45 µm filter. The protein was affinity-purified by immobilized metal-affinity chromatography on a 5 ml HisTrap FF Crude column (GE Healthcare). The eluted fractions containing protein were pooled, concentrated and further purified by size-exclusion chromatography on a HiLoad 16/60 Superdex 200 column (GE Healthcare) equilibrated in 20 m*M* Tris–HCl pH 7.5, 150 m*M* NaCl, 0.1 m*M* ADP, 2 m*M* DTT. Eluted peak fractions were pooled, concentrated to 7.7 mg ml^−1^ and stored at −80°C.

### 2.2. Cryo-EM imaging of KIF5A motor domain-decorated, GMPCPP-stabilized microtubules

To generate GMPCPP-stabilized microtubules, purified calf brain tubulin was resuspended at 4 mg ml^−1^ on ice using BRB80 buffer (80 m*M* PIPES, 1 m*M* EGTA, 1 m*M* MgCl_2_ pH 6.8) supplemented with 0.5 m*M* GMPCPP (Jena Bioscience). The tubulin solution was incubated on ice for 5 min and then centrifuged for 10 min at 16 800*g* at 4°C. The supernatant was transferred to a fresh tube and placed on a 37°C heat block for 40 min. The GMPCPP-stabilized microtubule solution was snap-frozen in liquid nitrogen and stored at −80°C until use.

To generate cryo-EM grid specimens containing KIF5A motor domain-decorated, GMPCPP-stabilized microtubules, freshly thawed KIF5A motor domain (7.7 mg ml^−1^) was diluted with BRB80 to 2 mg ml^−1^ KIF5A, supplemented with 5 m*M* AMPPCP and incubated for 30 min on ice. Meanwhile, GMPCPP-stabilized microtubules were thawed and warmed for 10 min at 37°C, followed by centrifugation at 16 800*g* for 5 min at 25°C. The supernatant was discarded and the microtubule pellet was resuspended in warm BRB80 buffer to a final tubulin concentration of 1.33 mg ml^−1^. 3.5 µl of the stabilized microtubule solution was pipetted onto a glow-discharged Quantifoil grid (R1.2/1.3, Cu, 200 mesh) and incubated for 60 s at room temperature. The grid was blotted by hand and 3 µl of the KIF5A–AMPCPP solution was added, and the grid was transferred to a Vitrobot Mark IV (Thermo Fisher Scientific) at 100% humidity and 25°C, and incubated for a further 60 s. Grids were blotted for 1 s with a blot force of 2, plunge-frozen in liquid ethane and stored in liquid nitrogen until data collection.

Data were acquired at the BioEM facility of the University of Basel on a Titan Krios electron microscope at 300 keV (Thermo Fisher) with a GIF Quantum LS Imaging filter (20 eV slit width) and a K2 Summit electron-counting direct detection camera (Gatan). 1676 movies were recorded at a magnification of 78 125×, corresponding to a pixel size of 0.64 Å. The total dose per movie was 65.0 e^−^ Å^−2^, fractionated over 62 frames. Data-collection statistics are also provided in Supplementary Table S1.

### 2.3. Cryo-EM processing of KIF5A motor domain-decorated, GMPCPP-stabilized microtubules

Data processing involved the following steps, as outlined for the *MiCSPARC* pipeline.

Movies underwent beam-induced motion correction and CTF estimation using *CryoSPARC*’s patch-based methods.

A rough template for particle picking was generated using *CryoSPARC*’s filament tracer, with 360 Å filament diameter and 82 Å separation distance between segments, corresponding to the heterodimeric repeat distance of α/β-tubulin. These were extracted and underwent 2D classification to select classes with a single clear tube in the 2D average, excluding picks with poor alignment, no or multiple tubes.

To generate a more complete and more correctly assigned set of filament picks, these initial picks were used to guide an extrapolation algorithm to sample potential filament positions across the micrographs. To account for misassignments of filament ID, particularly in the case of densely packed or overlapping filaments, existing filament groups were first split at particles with an in-plane rotation change greater than 10° from the preceding particle. Subsequently, a linear best fit was determined on a per-filament group basis, modeling the ideally straight arrangement of single-particle microtubule preparations. In cases where this resulted in significant variation against more than 10% of the initial picks in the filament, extrapolation was instead performed using a quadratic best fit, modeling a smooth, continuous curve while minimizing the chances of overfitting compared with higher order approximations. New extrapolated particle positions were again extracted and processed by 2D classification as described previously.

To create synthetic references from our data for protofilament sorting, a subset of particles was fed into six different helical refinement jobs with theoretical helical parameters corresponding to potential protofilament numbers in the dataset (protofilament number–start number: 11–3, 12–3, 13–3, 14–3, 15–4 and 16–4). The corresponding rise and twist for each helix is outlined in Section S1. After visual inspection of all helical refinement maps in *ChimeraX*, particles from the 14–3 map were selected for subsequent processing based on its well resolved tubulins and KIF5A decoration. Particles were symmetry-expanded using theoretical helical parameters for a 14–3 microtubule, centered on a single protofilament, refined and subjected to signal subtraction. This centered proto­filament reconstruction was then used to generate a series of volumes via *MiCSPARC*’s reference-generation script (Section S1).

The resulting volumes were used as references for microtubule architecture sorting by one round of heterogeneous refinement. Similarly to *MiRPv*2 (Cook *et al.*, 2020 ▸), class assignment within filament groups was ‘smoothened’, assigning each particle the modal value of the surrounding seven particles. Stretches of filament where over 70% of the particles had been assigned a different class to the rest of the filament were reassigned into their own filament groups, while lower confidence differences in assignment were unified to match the rest of the microtubule. Particles corresponding to the 14-protofilament, 3-start microtubule, which made up 83% of the dataset, were selected for further refinement.

To perform rough alignment of the microtubule, the particles underwent helical reconstruction with non-uniform refinement using the theoretical helical parameters (rise 8.79 Å, twist −25.7°) and global and local CTF refinement, and were finally reconstructed using helical refinement with no helical symmetry applied.

The angles in the plane of the micrograph (ψ) and the microtubule axial plane (φ) were corrected alongside local refinements. ψ angles were first unified within filaments by quadratic fit through particles with angles within ±10° of the modal ψ angle. To account for the somewhat random assignment of φ angles following helical refinement, the fit of 14 parallel linear models (corresponding to each protofilament) was optimized and the line of modal agreement was taken as the unifying value, ensuring that each particle was oriented such that each protofilament would be averaged in the same position as its neighbors. After refining the particles and performing an additional round of φ unification, the resulting particles were again refined and then symmetry-expanded 14-fold using the refined helical parameters and subjected to a final local refinement without recentering.

Following rough alignment of the whole microtubule, a single protofilament was selected and masked using the *ChimeraXSEGGER* tool (Pettersen *et al.*, 2021 ▸). In particular, the protofilament with the most mixed population of decorator registers was selected both to ensure that a tight mask did not cause any decorator signal to be removed during signal subtraction and to provide adequate population sizes of both registers for register-correction 3D classification.

The resulting volume and particles were shifted to be centered on a selected protofilament and the box size was restricted to exclude the opposite side of the microtubule. Particles were signal-subtracted to remove neighboring protofilaments and locally refined to produce a roughly aligned single-protofilament average, resulting in relatively high-resolution density for the tubulin heterodimers but poorer density for the decorator due to the combined occupancy of registers.

To separate the registers of the aligned protofilament, particles first underwent 3D classification with ten classes in *CryoSPARC*’s ‘simple’ initialization mode, with the filter resolution set to 12 Å to force the classification to focus on the large decorator density. Classes with the strongest density of the KIF5A motor domain corresponding to particles of the same register were locally refined together to produce two references of clear register difference.

These two references were used in a second round of 3D classification to separate the entire particle set into one of the two register classes. Each class was locally refined separately, and the class with worse reported resolution was chosen to be shifted by 41 Å (corresponding to a tubulin monomer) to align the register with the other class. The particles were finally combined, locally refined and underwent duplicate removal, global and local CTF refinement to produce a final high-resolution average of a single register-corrected protofilament.

To produce a reconstruction of the whole microtubule, the separation of protofilament registers was restored using *CryoSPARC*’s heterogeneous reconstruction while retaining the refined per-particle CTF information.

To determine the seam position, protofilaments were grouped into sets produced from the same symmetry-expanded particle, representing protofilaments of the same ‘layer’ within the microtubule, and scored on the likelihood that they laid at the seam position of that layer based on the registers of the surrounding protofilaments. This process was repeated along each microtubule and averaged per microtubule, producing a consensus seam position. The protofilament particles were un-symmetry-expanded by only retaining particles at the seam position.

Previous recentering and box-size reduction to focus on single protofilaments were reverted to reconstruct a volume of the whole microtubule. Particles were locally refined and underwent duplicate removal followed by global and local CTF refinement to produce a final high-resolution average of the 14–3 seam-resolved KIF5A-decorated GMPCPP-stabilized microtubule.

### 2.4. Cryo-EM imaging of undecorated GDP microtubules

To generate spontaneously polymerized microtubules, 25 µ*M* porcine brain tubulin in BRB80 buffer was supplemented with 1 m*M* GTP and 0.05%(*v*/*v*) NP-40 (Thermo Fisher Scientific) and was incubated at 37°C for 15 min. 3.5 µl microtubule solution was then applied onto a glow-discharged (25 mA, 30 s) C-flat holey thick carbon grid (R1.2/1.3, Cu, 300 mesh). After 30 s incubation in a Vitrobot (FEI–Thermo Fisher) at 37°C and 100% humidity, the grid was blotted for 4 s using filter paper (Ted Pella), plunge-frozen in liquid ethane and stored in liquid nitrogen until data collection.

5004 movies were collected using a Gatan K3 in CDS mode and a slit width of 15–20 eV on a GIF BioQuantum energy filter on a TFS Titan Krios G3i (2) FEG of the ScopeM facility at ETH, Zürich, Switzerland. Automatic data collection was performed using ‘Faster acquisition mode’ in *EPU* (Thermo Fisher Scientific). The dataset was collected at 81 000× magnification with a pixel size of 1.067 Å using counting mode. The total electron dose was 80 e^−^ Å^−2^ over 40 frames with a defocus range varying between −2.8 and −1.2 µm.

### 2.5. Cryo-EM processing of undecorated GDP microtubules

Data processing of undecorated microtubules proceeded in a similar fashion as outlined above for the decorated dataset, with some specific differences in parameters to adapt the process for the lack of an easily resolvable register marker.

Initial micrograph processing was performed as above, except that micrographs were additionally curated for good CTF estimates in *CryoSPARC*. For the filament tracer, a filament diameter of 280 Å was used, adjusting to the smaller visual diameter of the microtubule due to the lack of a decorator protein. Particle picking and extrapolation, protofilament number sorting and rough microtubule alignment otherwise proceeded as described above.

During protofilament number sorting, a mixed population of 13- and 14-protofilament microtubules was found. These underwent rough alignment, symmetry expansion and signal subtraction to expose single protofilaments separately, but protofilament particles from microtubules of both protofilament numbers were combined prior to register correction and refinement to allow a consensus refinement of the highest resolution.

Due to the lack of a resolvable difference in register at lower resolutions, 3D classification for register correction proceeded with a filter resolution of 4 Å, allowing the difference in the density of the S9–S10 loop between α- and β-tubulin to be somewhat resolved and classified against. To aid this, a mask around a single central tubulin monomer was also used during classification.

Unlike in the decorated dataset, ‘*ab initio*’ 3D classification only produced a strong average for one register of the protofilament. To circumvent this, a reference for the second register was created by recentering the volume by 41 Å using *CryoSPARC*’s volume-alignment tool, and further 3D classification of the two protofilament registers proceeded as previously using these references.

Refinement of the single protofilament, and reconstruction of the 13–3 microtubule (chosen due to having fewer particles for faster computational times), proceeded essentially as described for the KIF5A-decorated GMPCPP microtubule dataset.

### 2.6. Cryo-EM processing of MKLP2-decorated microtubules

*MiCSPARC*-based protofilament and microtubule reconstructions of MKLP2-decorated microtubules (EMPIAR-10796; Cook *et al.*, 2020 ▸) were generated essentially as for the KIF5A-decorated microtubule dataset (Supplementary Figs. S6*b* and S6*d*).

### 2.7. Model building

The KIF5A and tubulin models were generated based on an *AlphaFold*3 (Abramson *et al.*, 2024 ▸) prediction using the sequences of human KIF5A (UniProt accession ID Q12840), bovine tubulin α1B (P81947), bovine tubulin β2B (Q6B856), porcine tubulin α1A (P02550) and porcine tubulin β (P02554). The predicted structures were docked into their respective consensus maps using *ChimeraX* and subsequently refined manually in *Coot* (Emsley *et al.*, 2010 ▸). Final real-space refinement was performed in *Phenix* (Liebschner *et al.*, 2019 ▸). Model-refinement statistics for KIF5A-decorated and un­decorated tubulin are provided in Supplementary Table S2.

For MKLP2-decorated tubulin, since a refined model is not available, PDB entry 5nd4 was rigid-body fitted into the available *MiRPv*2-processed protofilament map (EMDB entry EMD-10131; Cook *et al.*, 2020 ▸) or into our *MiCSPARC* reconstruction of the same initial data (EMPIAR-10796; Cook *et al.*, 2020 ▸) using *ChimeraX* and both models were subsequently refined manually in *Coot* (Emsley *et al.*, 2010 ▸). Final real-space refinement was performed in *Phenix* (Liebschner *et al.*, 2019 ▸). Model-refinement statistics for MKLP2-decorated tubulin refined in either map are provided in Supplementary Table S4.

## 3. Results

### 3.1. Developing *MiCSPARC* to reconstruct a kinesin-1 motor domain-decorated, GMPCPP-stabilized microtubule

**Figure 1:**
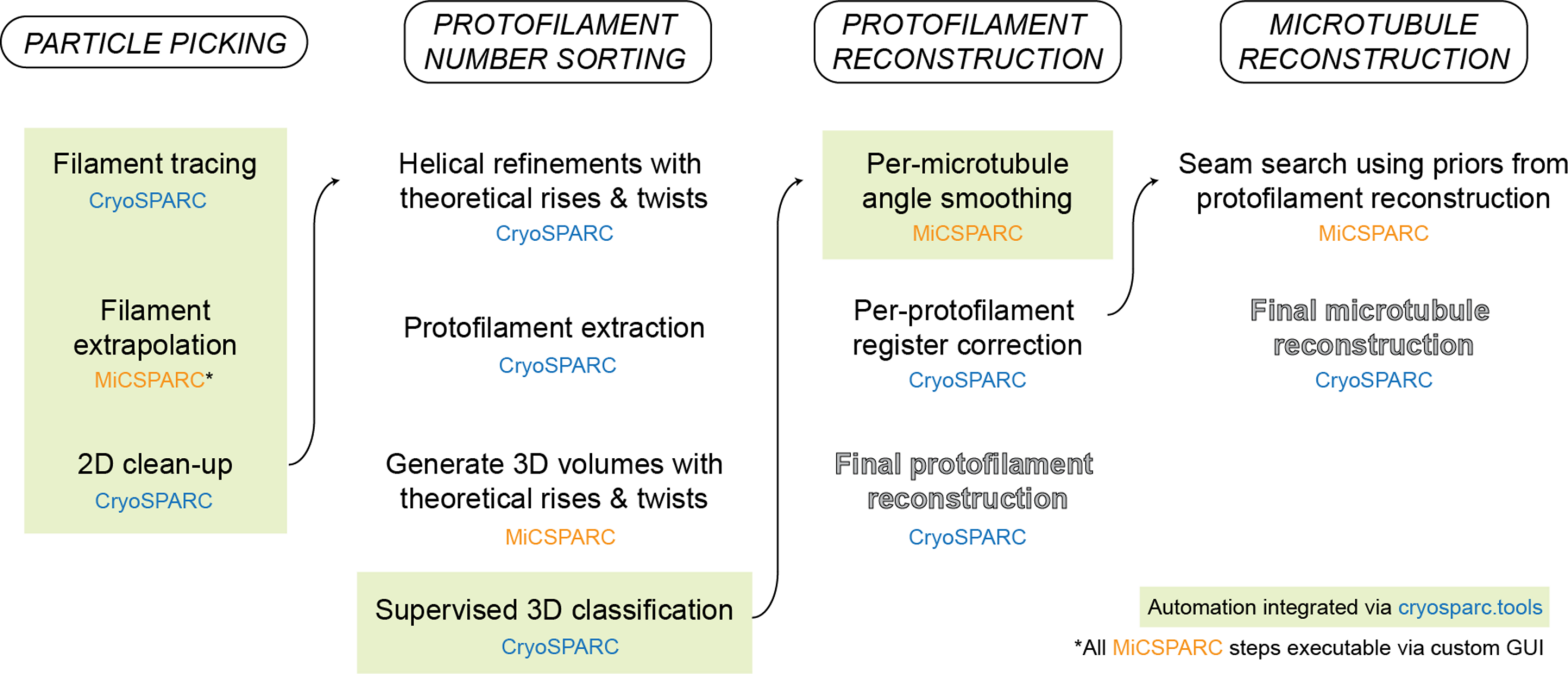
Schematic overview of the *MiCSPARC* processing pipeline.

We developed *MiCSPARC* (Fig. 1 ▸ and Section S1) using a test dataset of guanosine-5′-[(α,β)-methyleno]triphosphate (GMPCPP)-stabilized microtubules decorated with the microtubule-binding motor domain of KIF5A (Supplementary Fig. S1*a* and Table S1), a member of the kinesin-1 protein family. Microtubules decorated with kinesin motor domains are typically easier to align and average than undecorated filaments (Alushin *et al.*, 2014 ▸), and the relatively large kinesin motor domain acts as a fiducial that helps to localize particle positions relative to the seam in downstream processing steps (Ti *et al.*, 2018 ▸).

We first optimized the particle-picking step, which normally involves tracing the microtubule and extracting overlapping segments every ∼8 nm as 2D particles. Manual selection of microtubule segment start and end points, as previously described (Cook *et al.*, 2020 ▸), is a time-consuming, tedious process that does not typically accommodate curved filament picking and can be prone to user bias. *CryoSPARC*’s filament tracer reliably picks microtubules without significant user intervention, but it also tends to incorrectly assign particles from different filaments into the same filament. Moreover, it can miss portions of filaments entirely, particularly near the edges of micrographs or in densely populated regions (Supplementary Fig. S2*a*).

To address these challenges, we developed a script that extrapolates coordinates from the filament-tracer tool in *CryoSPARC* to more completely select and keep track of microtubule filaments. The script incorporates both linear and quadratic curve fitting to account for slight, naturally occurring bends in microtubules imaged by cryo-EM. By iteratively comparing best-fit models of each filament with particles picked across all filaments, proximal particles are dynamically reassigned, mitigating the effects of incorrect assignments from *CryoSPARC*’s filament-tracer tool. Additionally, generating a typically larger number of starting particles using this approach also alleviates the need to generate locally averaged ‘super-particles’, which is presently required by other methods for accurate initial particle alignments (Zhang & Nogales, 2015 ▸; Cook *et al.*, 2020 ▸).

Next, we focused on the challenge of generating 3D references for supervised classification. In current microtubule-reconstruction pipelines, 3D references are either used directly from ‘standard’ datasets of undecorated and decorated microtubules (for example, Sui & Downing, 2010 ▸; Cook *et al.*, 2020 ▸), or else synthetic ‘decorated’ references mimicking the addition of the binding protein need to be generated (Zhang & Nogales, 2015 ▸), which requires accurate knowledge of the structure of the decorator and its binding mode on the tubulin lattice. In our hands, synthetic or *a priori* references can work when the shape and location of the binder are already known but will tend to fail without a priori knowledge. Even with such knowledge, slight mismatches between reference and real structures due to, for example, unexpected helical supertwists (Zhang *et al.*, 2015 ▸, 2018 ▸) can lead to nonconverging 3D refinements.

**Figure 2:**
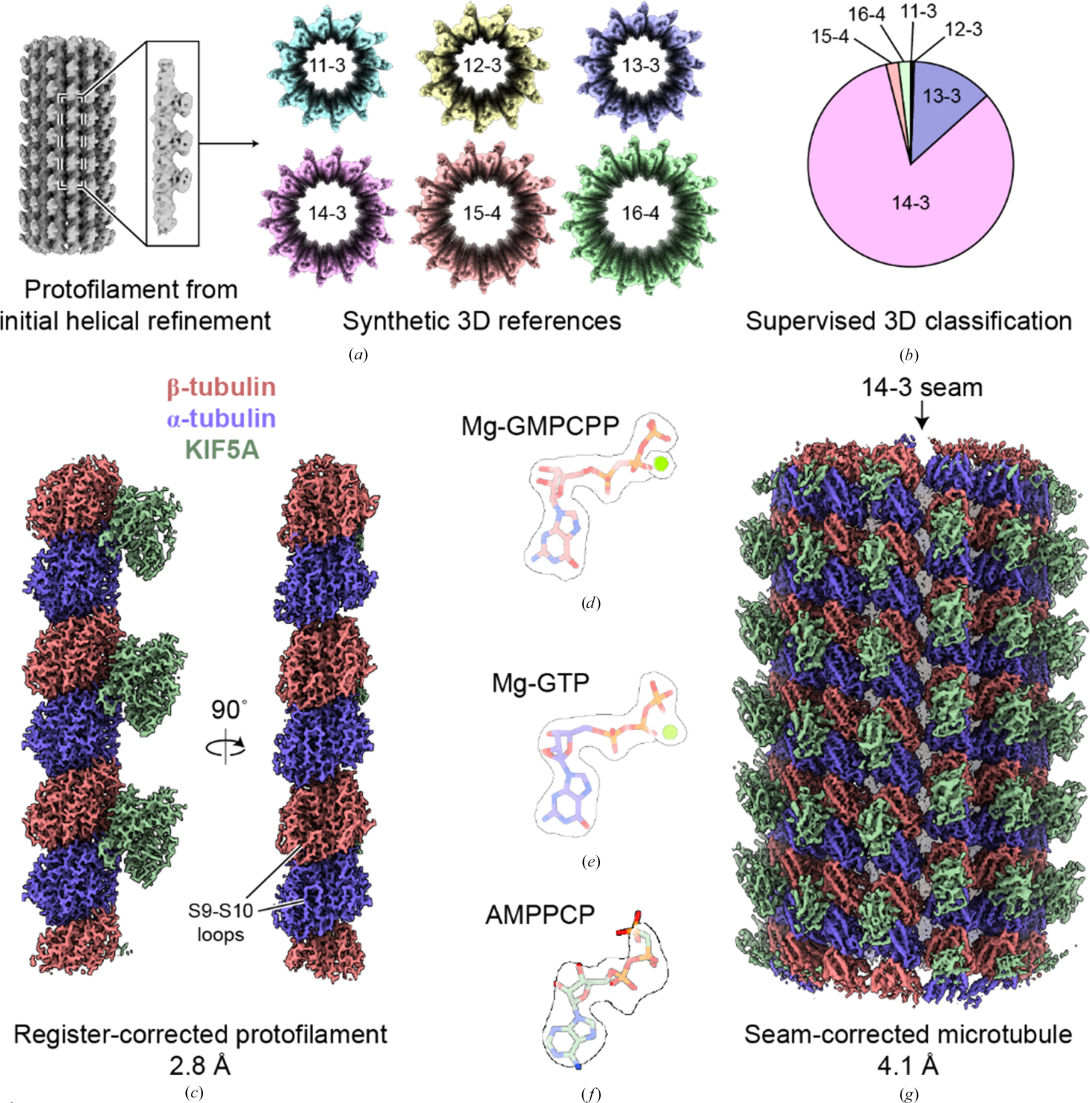
*MiCSPARC* reconstruction of a KIF5A motor domain-decorated, GMPCPP-stabilized microtubule. (*a*) Initial rough signal-subtracted reconstruction of a single KIF5A-decorated protofilament (left), which was then used to computationally generate synthetic 3D references for supervised 3D classification (right; colored reconstructions; protofilament number and helical ‘start’ numbers are labeled). (*b*) The resulting distribution from supervised 3D classification and 3D class ‘smoothing’ of the KIF5A-decorated microtubule dataset using the references in (*a*). (*c*) Two views of a final *MiCSPARC* refinement of a single KIF5A-decorated protofilament after symmetry expansion and α/β-tubulin register correction. Obvious differences in α-tubulin versus β-tubulin S9–S10 loops are indicated. The map was post-processed using *EMReady* (He *et al.*, 2023 ▸). (*d*) Mg-GMPCPP in β-tubulin (stick representation) in the corresponding density (transparent surface representation). (*e*) Mg-GTP in α-tubulin (stick representation) in the corresponding density (transparent surface representation). (*f*) AMPPCP in KIF5A (stick representation) in the corresponding density (transparent surface representation). Densities in (*d*)–(*f*) are thresholded at the same levels. (*g*) Seam view of the seam-corrected, 14-protofilament and 3-start microtubule reconstruction obtained using *MiCSPARC*. The location of the seam is indicated by an arrow.

In *MiCSPARC*, we dealt with these challenges by reconstructing (after symmetry expansion and signal subtraction) a single protofilament from an initial reference-free helical refinement of all picked particles. This protofilament was then helically replicated to generate a set of synthetic references with any desired theoretical helical parameters (Fig. 2 ▸*a*). Synthetic references were then used for supervised heterogeneous refinement in *CryoSPARC*. This resulted in a robust sorting of microtubule architectures (Fig. 2 ▸*b*), as judged by the dominant microtubule architecture in the kinesin-decorated dataset being 14 protofilaments with a 3-start helix (14–3), which is expected for microtubules polymerized in the presence of GMPCPP (Hyman *et al.*, 1992 ▸). Using information from this supervised classification, *MiCSPARC* then employs a smoothing algorithm to assign a protofilament number along each picked microtubule (Cook *et al.*, 2020 ▸), which is also needed to generate accurate seam-corrected microtubule reconstructions below.

The subsequent processing steps in *MiCSPARC* have two main goals: (i) protofilament reconstruction, which allows higher resolutions to be reached locally but also unifies incorrectly assigned Euler angles from global refinements, and (ii) the generation of a seam-corrected microtubule reconstruction, which uses the alignment information determined in (i). For protofilament reconstruction [goal (i)], we first assigned each filament to a specific microtubule architecture based on whether the majority of its particles classified to the same architecture, analogous to previous methods (Cook *et al.*, 2020 ▸). Microtubule particles from a given protofilament number from the 3D classification step were first refined, and per-microtubule in-plane (ψ) rotation angles were then unified, following previously described methods (Cook *et al.*, 2020 ▸). ψ-unified particles were re-imported and locally refined in *CryoSPARC*. Angles representing rotations around the filament axis (φ) were then also iteratively unified on a per-microtubule basis, using a modification of previous methods (Cook *et al.*, 2020 ▸; see Section 2).

After a final *C*1 refinement using the updated priors, each particle within a given 3D class was next symmetry-expanded using the previous helical refinement outputs, and a single protofilament was masked and again subjected to a local refinement. 3D classification without alignment in *Cryo­SPARC* was employed to identify two protofilament maps that are translated by one tubulin monomer relative to one another; this is identifiable by the shifted presence of the kinesin motor-domain decorator. These maps were used as references for supervised 3D classification of all symmetry-expanded protofilament particles. Alignment of one of the resulting classes to the other followed by final refinement steps resulted in a 2.8 Å resolution reconstruction of the proto­filament, in which all particles contain the correct α/β-tubulin register (Fig. 2 ▸*c*, Supplementary Figs. S1*a*–S1*f* and Supplementary Table S1). The quality of the reconstruction was also sufficient to identify all expected nucleotide states in the kinesin motor domain, α-tubulin and β-tubulin, including coordinated magnesium ions representing pre-hydrolysis states (Figs. 2 ▸*d*–2 ▸*f*, Supplementary Figs. S3*a* and S3*b* and Supplementary Table S2).

To reconstruct the seam-corrected, decorated microtubule [goal (ii)], the sets of particles classified during α/β-tubulin register correction above were used to determine the position of the microtubule seam (Supplementary Fig. S2*b*). In principle, the seam is the position at which the protofilament register changes. Thus, the position of the seam can be determined where consecutive symmetry-expanded and well aligned particles switch from occupying one register class to the other within the same microtubule filament. However, as the 3D classification during α/β-tubulin register correction is not perfect, corrective measures can also be applied to improve the confidence of the assignment. We therefore scored each position on the likelihood that it is the seam position, based on the registers of neighboring protofilaments. The score increases if the protofilaments on one side of the position have been assigned to the same register and to a different register to the protofilaments on the opposite side. Due to the previous φ-angle correction, the order of protofilaments in each image along the microtubule can be assumed to be the same. Thus, the seam-likelihood scores for each position can be averaged along the microtubule to give a more robust estimation of the most likely seam position. The particles at the most likely seam position are then returned to be extracted and refined as the seam-corrected microtubule.

For the KIF5A-decorated dataset, this procedure generated a 4.2 Å resolution reconstruction of the decorated microtubule (Fig. 2 ▸*g*, Supplementary Figs. S3*c*–S3*g* and Supplementary Table S1), in which the seam of the 14 protofilament, 3-start microtubule can be identified by an obvious shift in KIF5A decorating densities, as well as differences in the S9–S10 loops of α- and β-tubulin. Together, these results demonstrate the effectiveness of *MiCSPARC* in generating high-resolution 3D reconstructions of α/β-tubulin bound to decorating fragments at the level of both the protofilament and the complete microtubule polymer.

### 3.2. *MiCSPARC* reconstruction of an undecorated GDP microtubule

Microtubule-binding proteins typically recognize a feature of the α/β-tubulin heterodimer. For example, the kinesin-1 motor domain binds specifically to the interface between α- and β-tubulin within the tubulin heterodimer. This facilitates identification of the α/β-tubulin register along each protofilament and therefore correction for the seam, which is why kinesin motor-domain decoration has been used extensively in many previous microtubule structural studies (Alushin *et al.*, 2014 ▸; Ti *et al.*, 2018 ▸). However, it has also been reported that certain microtubule-binding domains, including those from kinesins, can induce structural changes in the tubulin lattice (Lacey *et al.*, 2019 ▸; Zhang *et al.*, 2018 ▸), potentially confounding the interpretation of tubulin properties from high-resolution decorated microtubule structures.

**Figure 3:**
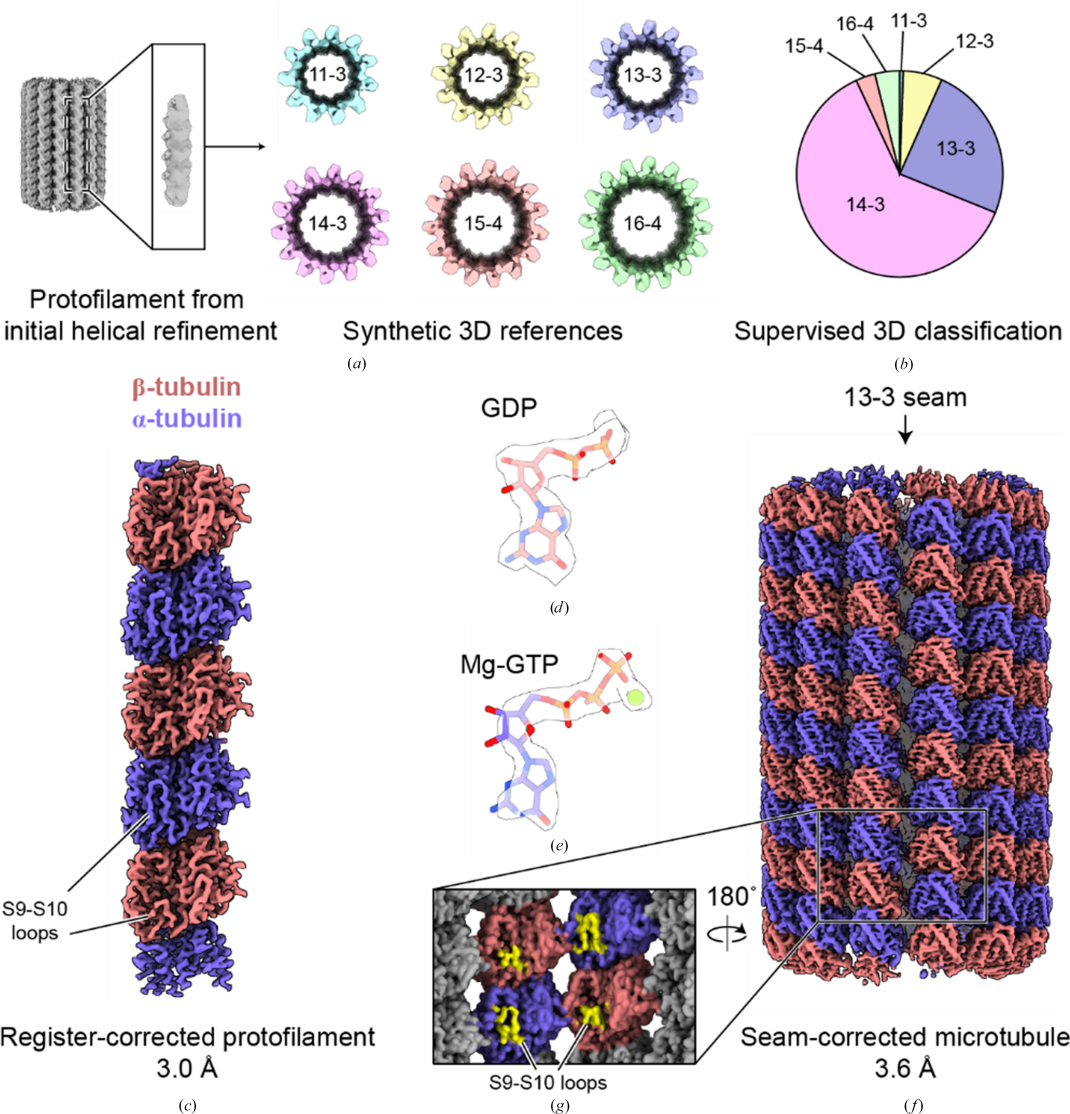
*MiCSPARC* reconstruction of an undecorated, dynamic microtubule. (*a*) Initial rough signal-subtracted reconstruction of a single protofilament (left), which was then used to computationally generate synthetic 3D references for supervised 3D classification (right; colored reconstructions; protofilament number and helical ‘start’ numbers are labeled). (*b*) Resulting distribution from supervised 3D classification and 3D class ‘smoothing’ of the undecorated microtubule dataset using the references in (*a*). (*c*) Lumenal view of a final *MiCSPARC* refinement of a single undecorated protofilament after symmetry expansion and α/β-tubulin register correction. Obvious differences in α-tubulin versus β-tubulin S9–S10 loops are indicated. The map was post-processed using *EMReady* (He *et al.*, 2023 ▸). (*d*) GDP in β-tubulin (stick representation) in the corresponding density (transparent surface representation). (*e*) Mg-GTP in α-tubulin (stick representation) in the corresponding density (transparent surface representation). (*f*) Seam view of the seam-corrected, 13-protofilament and 3-start microtubule reconstruction obtained using *MiCSPARC*. The location of the seam is indicated by an arrow. (*g*) Zoomed-in and rotated view of the seam of the microtubule reconstruction in (*f*). Differences in α-tubulin versus β-tubulin S9–S10 loops across the seam are indicated.

We therefore next asked whether *MiCSPARC* could be used to classify and seam-reconstruct a microtubule lacking any binding proteins. This presents a more difficult processing problem, due to the necessity of the subclassification of subtle differences between α- and β-tubulin, such as the S9–S10 loops, in two proteins with otherwise highly similar folds (Nogales *et al.*, 1995 ▸). We collected a cryo-EM dataset of microtubules polymerized in the presence of GTP (Supplementary Table S1), creating mainly GDP-containing lattices (Alushin *et al.*, 2014 ▸), and used *MiCSPARC*’s automated filament-tracing, reference-generation and supervised-classification pipelines to classify the microtubules into distinct lattice architectures. Consistent with previous findings, *MiCSPARC* sorted our spontaneously polymerized microtubule images into predominantly 14–3 and 13–3 protofilament architectures (Figs. 3 ▸*a* and 3 ▸*b*). Gratifyingly, we were next able to use *MiCSPARC* to distinguish between α- and β-tubulin and generate a reconstruction of an α/β-tubulin protofilament at 3.0 Å resolution (Fig. 3 ▸*c*, Supplementary Figs. S4*a*–S4*f* and Supplementary Table S1). Consistent with current models (Alushin *et al.*, 2014 ▸; Hyman *et al.*, 1992 ▸), the resulting map shows that α/β-tubulin is in a ‘compacted’ microtubule lattice bound conformation. Further, at these resolutions nucleotide features can be clearly distinguished, revealing a magnesium ion and GTP at the nucleotide-binding site of α-tubulin, while β-tubulin exhibits only GDP (Figs. 3 ▸*d* and 3 ▸*e*, Supplementary Figs. S5*a* and S5*b* and Supplementary Table S2), consistent with biochemical studies of α/β-tubulin (Menéndez *et al.*, 1998 ▸).

Re-extracting these aligned particles to reconstruct the microtubule (3.5 Å resolution; Figs. 3 ▸*f* and 3 ▸*g*, Supplementary Figs. S5*c*–S4*g* and Supplementary Table S1) revealed well defined S9–S10 loops in α- and β-tubulin and heterotypic contacts at the seam. This suggests that *MiCSPARC* accurately detects the seam of even undecorated microtubules, and therefore it accurately assigns the φ angles in the protofilament reconstruction step via a possibly more robust algorithm compared with previous approaches (see Section 2; Cook *et al.*, 2020 ▸).

Lastly, and to more directly compare the quality of *MiCSPARC* reconstructions with those of previous methods, we used it to generate protofilament and microtubule reconstructions of MKLP2-decorated microtubules from a publicly available dataset (EMPIAR-10796; Cook *et al.*, 2020 ▸). These reconstructions display similar overall resolutions and slightly better per-residue model quality scores compared with a previously described reconstruction generated using *MiRPv*2 (Supplementary Figs. S6 and S7 and Supplementary Tables S3 and S4; Cook *et al.*, 2020 ▸; Atherton *et al.*, 2017 ▸). Altogether, these results demonstrate that *MiCSPARC* can reliably generate high-resolution, seam-corrected reconstructions of not only decorated microtubules, but also more challenging undecorated microtubule specimens.

## 4. Discussion

We have described *MiCSPARC*, a cryo-EM processing pipeline for microtubules that leverages the *CryoSPARC* SPA platform. The key advantages of *MiCSPARC* over previous methods include (i) automated particle picking, (ii) *a priori*3D reference generation for supervised 3D classification of different microtubule-architecture types, (iii) robust reconstructions of undecorated or sparsely decorated microtubules with correctly estimated seam positions and (iv) enhancements in reconstruction throughput due to *CryoSPARC*’s faster refinement times compared with the alternatives (Punjani *et al.*, 2017 ▸), its user-friendly interface and its job-queueing system. While *MiCSPARC* serves as a reliable starting point for reconstructing microtubules from cryo-EM images, achieving optimal reconstructions may still require additional refinement steps either in *CryoSPARC* itself or with other established cryo-EM SPA software packages such as *RELION* (Scheres, 2012 ▸).

*MiCSPARC* has several areas for improvement in future versions. These could include further optimization of automated particle picking and 3D classification during microtubule architecture sorting, as well as α/β-tubulin register correction and seam correction. Moreover, challenges persist in handling decorated filaments exhibiting partial or flexible binding to the microtubule lattice, and the 3D reference-generation process still requires users to suggest microtubule-architecture types, which may lead to misclassification of unusual helical supertwists or lattice architectures.

Nevertheless, *MiCSPARC* is a robust method that enables the processing of microtubule specimens that may not meet traditional optimality criteria, thereby increasing reconstruction throughput. This may be particularly valuable for exploring nucleotide-state transitions in motor proteins and managing heterogeneities in decorator-binding modes. Factors such as mask choice and alignment quality during signal subtraction can influence the results, especially for varied decorators. To address this need for high throughput and ease of use, *MiCSPARC* also includes user-friendly GUI tools for visualizing angle-smoothing accuracy and seam reconstruction, while also facilitating integration with *CryoSPARC* projects, as well as automation of the earlier steps of the pipeline (Section S2). These tools have been made available to the academic community at https://github.com/wieczoreklab/MiCSPARC.

The regulation of microtubule polymerization is closely linked to structural dynamics in α/β-tubulin (reviewed in Brouhard & Rice, 2018 ▸). Yet the field lacks a comprehensive atomic-level view of how structural transitions that take place in α/β-tubulin during various stages of GTP hydrolysis influence microtubule nucleation, growth and disassembly. Additionally, local heterogeneities in microtubule lattice structure, particularly at the seam, are an intriguing target for structural investigations (Zhang *et al.*, 2018 ▸), but these features have been difficult to dissect with existing cryo-EM SPA methods. Alternative computational pipelines such as *MiCSPARC*, building on recent advances in SPA in cryo-EM, should provide a foundational suite of tools for dissecting these models at atomic resolution in the future.

## 5. Related literature

The following reference is cited in the supporting information for this article: Terashi *et al.* (2022 ▸).

## Supplementary Material

PDB reference: KIF5A-decorated GMPCPP microtubule, 9t17

PDB reference: undecorated GDP microtubule, 9t1d

EMDB reference: KIF5A-decorated GMPCPP protofilament, EMD-55425

EMDB reference: KIF5A-decorated 14–3 GMPCPP microtubule, EMD-55444

EMDB reference: undecorated GDP protofilament, EMD-55431

EMDB reference: undecorated 13–3 GDP microtubule, EMD-55440

Supplementary Figures and Tables, suggester MiiSPARC pipeline and MiCSPARC automation. DOI: 10.1107/S2059798326003062/zi5010sup1.pdf

Current MiCSPARC git repository in a zip folder. DOI: 10.1107/S2059798326003062/zi5010sup2.zip

## Data Availability

*MiCSPARC* scripts and detailed instructions are available at https://github.com/wieczoreklab/MiCSPARC. Structural models have been deposited in the PDB (KIF5A-decorated tubulin, PDB entry 9t17; undecorated GDP tubulin, PDB entry 9t1d). Cryo-EM maps have been deposited in the EMDB (KIF5A-decorated GMPCPP protofilament, EMDB entry EMD-55425; KIF5A-decorated 14–3 GMPCPP microtubule, EMDB entry EMD-55444; undecorated GDP protofilament, EMDB entry EMD-55431; undecorated 13–3 GDP microtubule, EMDB entry EMD-55440). Motion-corrected cryo-EM micrographs have been deposited to EMPIAR (KIF5A-decorated GMPCPP microtubules, EMPIAR-13372; undecorated GDP microtubules, EMPIAR-13367).
