# Supplementary material for: A cryo-EM processing pipeline for microtubules using CryoSPARC
Source: Acta Crystallogr D Struct Biol. 2026 Apr 27;82(Pt 5):411–20. doi: 10.1107/S2059798326003062 (PMC13133983; doi:10.1107/S2059798326003062)
Supplement: Supplementary file 1 [file d-82-00411-sup1.pdf]

STRUCTURAL
BIOLOGY

Volume 82 (2026)

Supporting information for article:

A cryo-EM processing pipeline for microtubules using *CryoSPARC*

Daniel Zhang, Hugo Muñoz-Hernández, Pavel Filipcik, Kushal Sejwal, Yixin Xu, Sung Ryul Choi, Michel O. Steinmetz and Michal Wieczorek

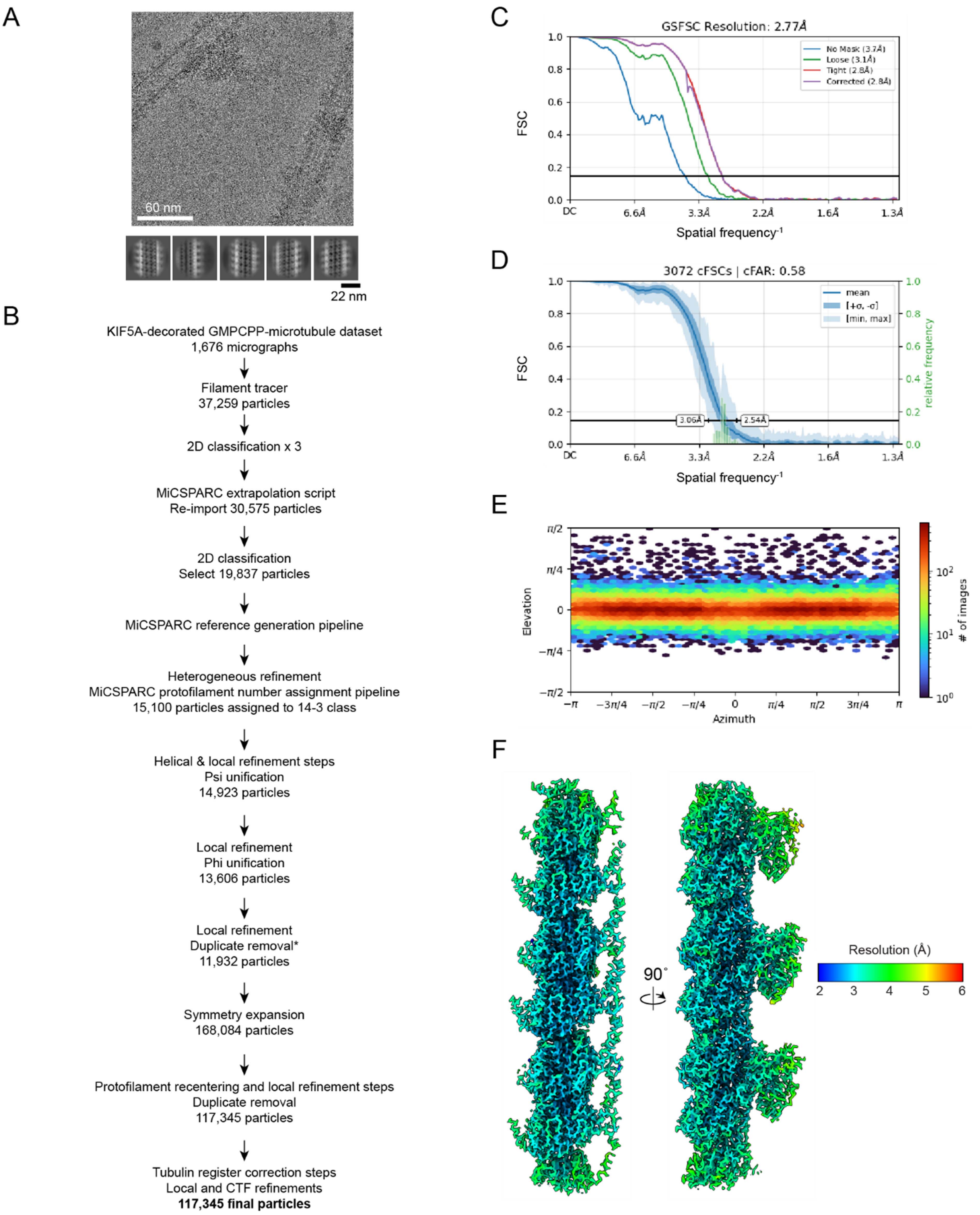
A
60 nm
22 nm
B
KIF5A-decorated GMPCPP-microtubule dataset
1,676 micrographs
Filament tracer
37,259 particles
2D classification x 3
MiCSPARC extrapolation script
Re-import 30,575 particles
2D classification
Select 19,837 particles
MiCSPARC reference generation pipeline
Heterogeneous refinement
MiCSPARC protofilament number assignment pipeline
15,100 particles assigned to 14-3 class
Helical & local refinement steps
Psi unification
14,923 particles
Local refinement
Phi unification
13,606 particles
Local refinement
Duplicate removal*
11,932 particles
Symmetry expansion
168,084 particles
Protofilament recentering and local refinement steps
Duplicate removal
117,345 particles
Tubulin register correction steps
Local and CTF refinements
117,345 final particles
C
GSFSC Resolution: 2.77Å
No Mask (3.7Å)
Loose (3.1Å)
Tight (2.8Å)
Corrected (2.8Å)
FSC
Spatial frequency⁻¹
D
3072 cFSCs | cFAR: 0.58
mean
[+σ, -σ]
[min, max]
3.06Å
2.54Å
FSC
relative frequency
Spatial frequency⁻¹
E
Elevation
Azimuth
# of images
F
90°
Resolution (Å)
2 3 4 5 6

Figure S1 Data processing of the KIF5A-decorated GMPCPP protofilament reconstruction. **A)** Top: example cryo-EM micrograph of KIF5A-decorated GMPCPP microtubules. Bottom: example 2D class averages obtained during particle cleanup. **B)** Workflow for processing the register-corrected protofilament reconstruction using MiCSPARC. **C)** CryoSPARC gold standard Fourier shell correlation (FSC) curve for the protofilament reconstruction obtained in B). **D)** CryoSPARC conical FSC Area Ratio (cFAR) plot for the protofilament reconstruction obtained in B). **E)** CryoSPARC particle orientation distribution for the protofilament reconstruction obtained in B). **F)** Lumenal and side views of the protofilament reconstruction obtained in B) coloured according to local resolution, as calculated in CryoSPARC. The map was postprocessed using EMReady (He et al., 2023).

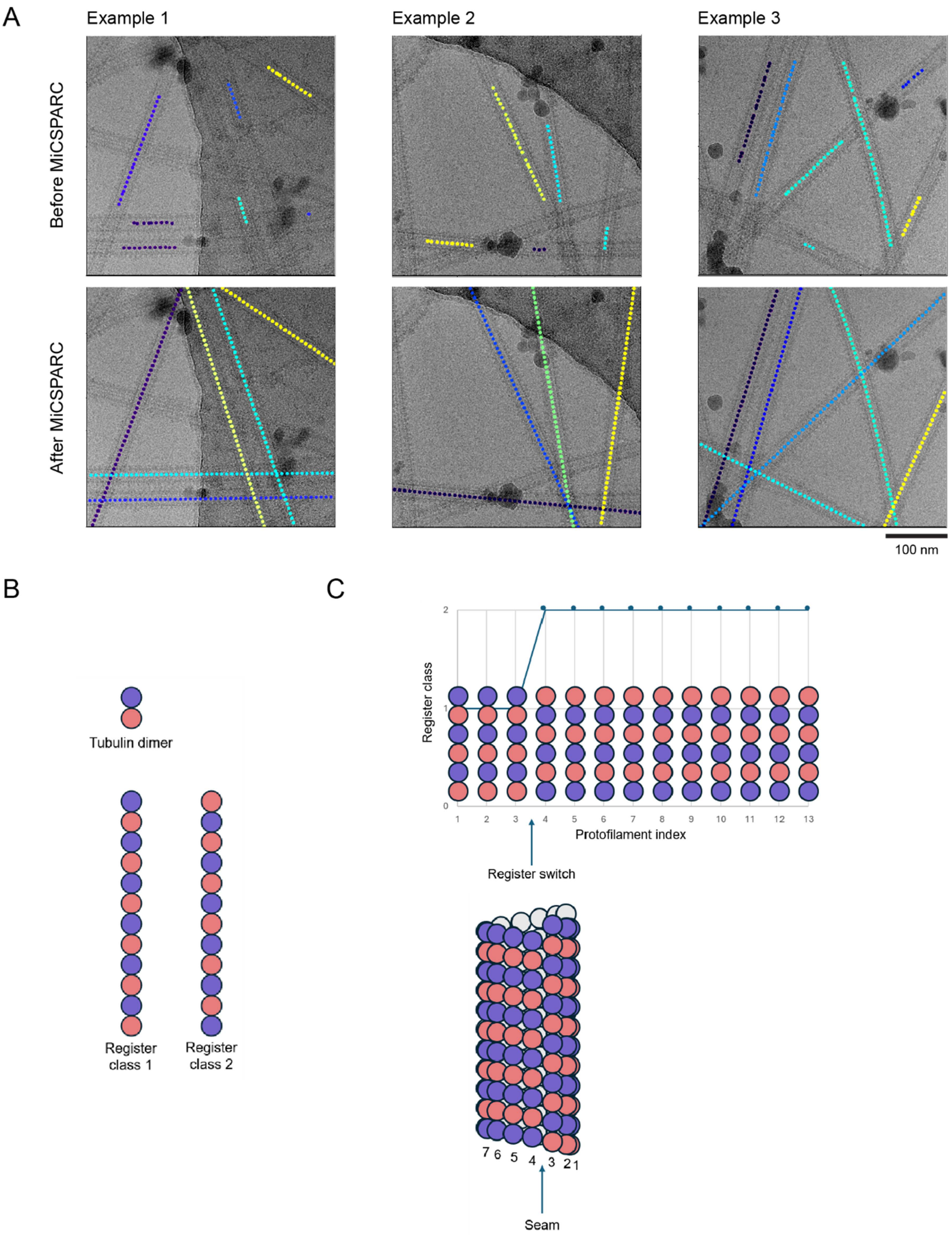
A
Example 1
Example 2
Example 3
Before MiCSPARC
After MiCSPARC
100 nm
B
Tubulin dimer
Register class 1
Register class 2
C
Register class
Protofilament index
Register switch
Seam

Figure S2 Filament tracing and seam correction in MiCSPARC. **A)** Three example outputs of particles picked by CryoSPARC's filament tracer tool (top row) compared with the resulting output after MiCSPARC extrapolation (bottom row). Particles are coloured according to reported filament IDs. **B)-C)** Schematic demonstrating the principles of seam finding by protofilament register. Two classes of protofilaments of opposing register are shown separated (**B**). The seam corresponds to the position around the microtubule at which the protofilament register flips (**C**). Thus, identifying the position of the change in register class for each symmetry-expanded particle set, and averaging along the microtubule using the conserved protofilament indices, allows the highest confidence position to be found along the length of the microtubule.

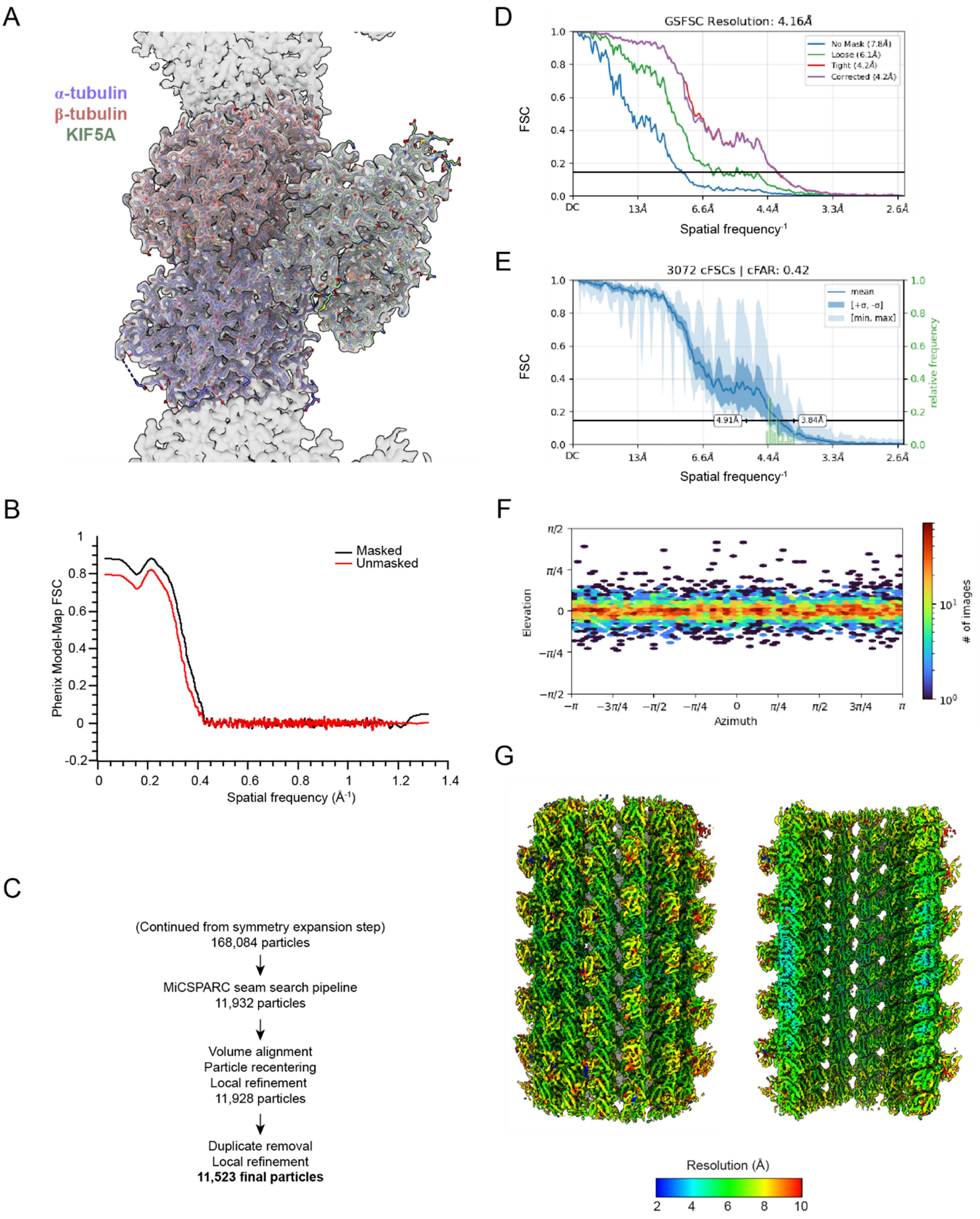
A
α-tubulin
β-tubulin
KIF5A
B
Masked
Unmasked
Phenix Model-Map FSC
Spatial frequency (Å⁻¹)
C
(Continued from symmetry expansion step)
168,084 particles
MiCSPARC seam search pipeline
11,932 particles
Volume alignment
Particle recentering
Local refinement
11,928 particles
Duplicate removal
Local refinement
11,523 final particles
D
GSFSC Resolution: 4.16Å
No Mask (7.8Å)
Loose (6.1Å)
Tight (4.2Å)
Corrected (4.2Å)
FSC
Spatial frequency⁻¹
E
3072 cFSCs | cFAR: 0.42
mean
[+σ, -σ]
[min, max]
4.91Å
3.84Å
relative frequency
FSC
Spatial frequency⁻¹
F
Elevation
Azimuth
# of images
G
Resolution (Å)
2
4
6
8
10

Figure S3 Data processing of the KIF5A-decorated GMPCPP microtubule reconstruction. **A)** Stick representation views of KIF5A-decorated α/β-tubulin model refined in the GMPCPP microtubule protofilament density map (transparent grey surface). Map was sharpened using EMReady (He *et al.*, 2023). **B)** Phenix map-model FSC curves for the KIF5A-decorated α/β-tubulin model refined in the GMPCPP microtubule protofilament density map. **C)** Workflow for processing the seam-corrected KIF5A-decorated 14-3 GMPCPP microtubule using MiCSPARC. **D)** CryoSPARC gold standard FSC curve for the microtubule reconstruction obtained in C). **E)** CryoSPARC cFAR plot for the protofilament reconstruction obtained in C). **F)** CryoSPARC particle orientation distribution for the protofilament reconstruction obtained in C). **G)** Lumenal and side views of the protofilament reconstruction obtained in B) coloured according to local resolution, as calculated in CryoSPARC. The map was postprocessed using EMReady (He et al., 2023).

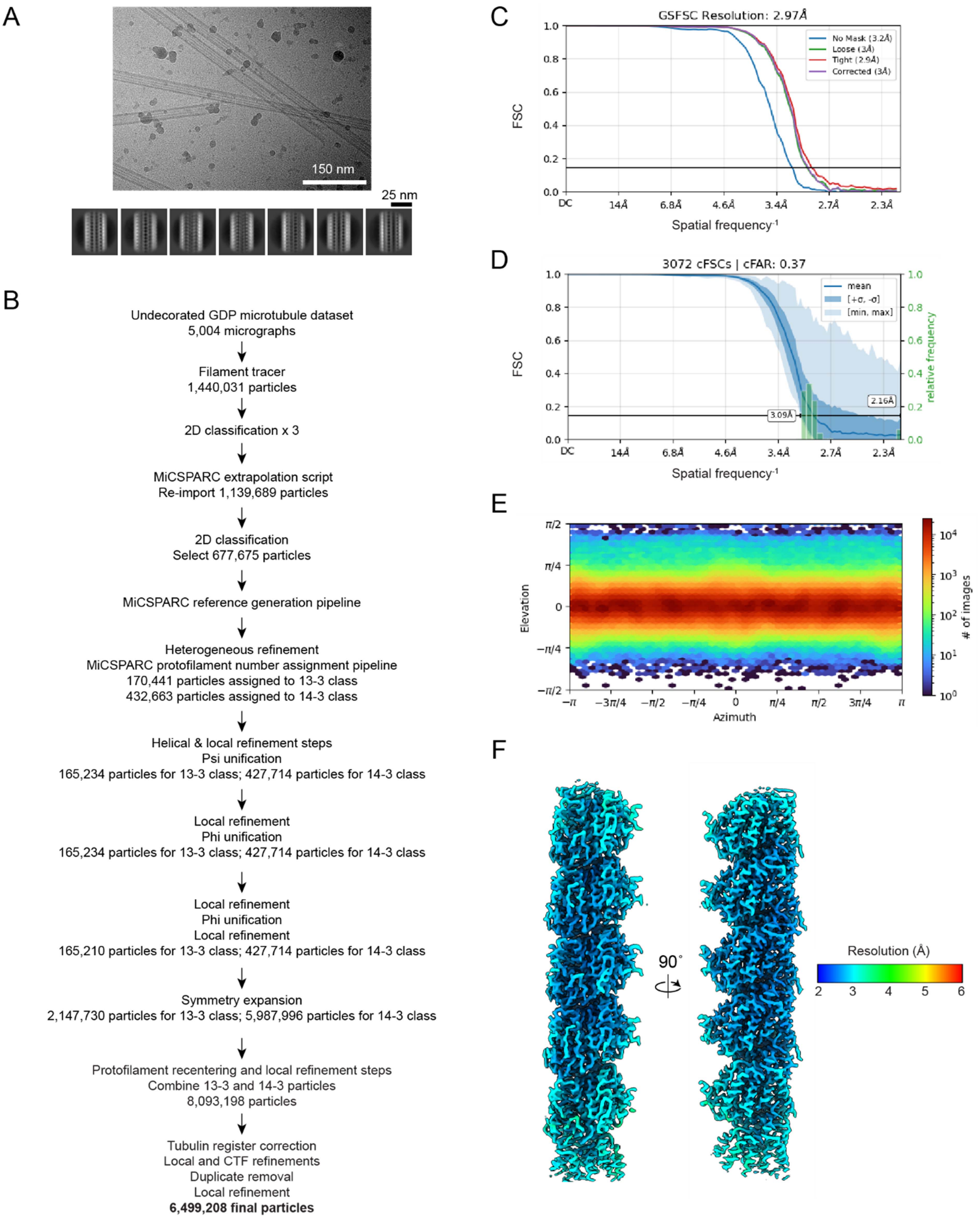
A
150 nm
25 nm
B
Undecorated GDP microtubule dataset
5,004 micrographs
Filament tracer
1,440,031 particles
2D classification x 3
MiCSPARC extrapolation script
Re-import 1,139,689 particles
2D classification
Select 677,675 particles
MiCSPARC reference generation pipeline
Heterogeneous refinement
MiCSPARC protofilament number assignment pipeline
170,441 particles assigned to 13-3 class
432,663 particles assigned to 14-3 class
Helical & local refinement steps
Psi unification
165,234 particles for 13-3 class; 427,714 particles for 14-3 class
Local refinement
Phi unification
165,234 particles for 13-3 class; 427,714 particles for 14-3 class
Local refinement
Phi unification
Local refinement
165,210 particles for 13-3 class; 427,714 particles for 14-3 class
Symmetry expansion
2,147,730 particles for 13-3 class; 5,987,996 particles for 14-3 class
Protofilament recentering and local refinement steps
Combine 13-3 and 14-3 particles
8,093,198 particles
Tubulin register correction
Local and CTF refinements
Duplicate removal
Local refinement
6,499,208 final particles
C
GSFSC Resolution: 2.97Å
No Mask (3.2Å)
Loose (3Å)
Tight (2.9Å)
Corrected (3Å)
FSC
Spatial frequency⁻¹
D
3072 cFSCs | cFAR: 0.37
mean
[+σ, -σ]
[min, max]
3.09Å
2.16Å
FSC
relative frequency
Spatial frequency⁻¹
E
Elevation
Azimuth
# of images
F
90°
Resolution (Å)
2 3 4 5 6

Figure S4 Data processing of the undecorated GDP protofilament reconstruction**. A)** Top: example cryo-EM micrograph of undecorated GDP microtubules. Bottom: example 2D class averages obtained during particle cleanup. **B)** Workflow for processing the register-corrected protofilament reconstruction using MiCSPARC. **C)** CryoSPARC gold standard Fourier shell correlation (FSC) curve for the protofilament reconstruction obtained in B). **D)** CryoSPARC conical FSC Area Ratio (cFAR) plot for the protofilament reconstruction obtained in B). **E)** CryoSPARC particle orientation distribution for the protofilament reconstruction obtained in B). **F)** Lumenal and side views of the protofilament reconstruction obtained in B) coloured according to local resolution, as calculated in CryoSPARC. The map was postprocessed using EMReady (He et al., 2023).

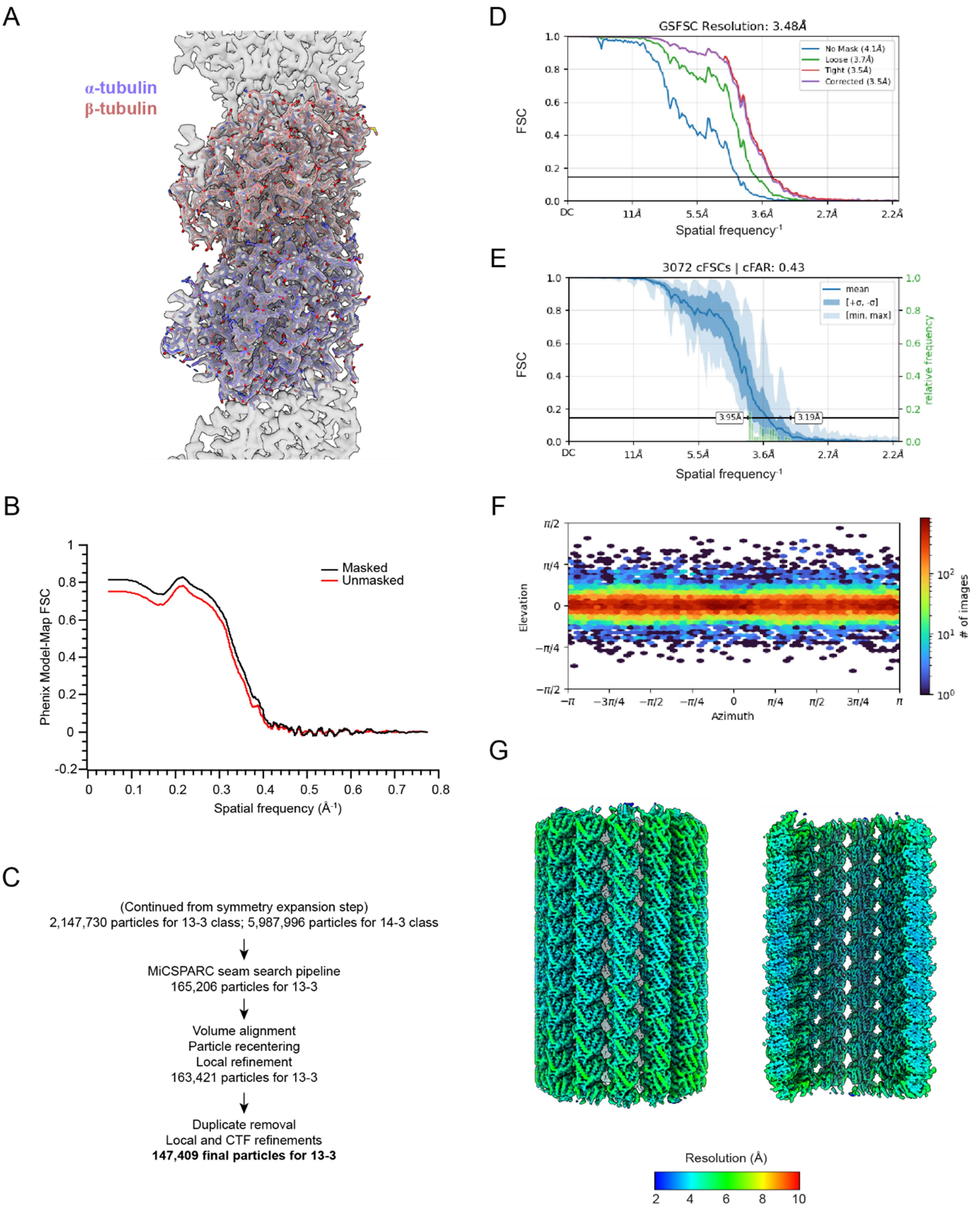
A
α-tubulin
β-tubulin
B
Phenix Model-Map FSC
Masked
Unmasked
Spatial frequency (Å-1)
C
(Continued from symmetry expansion step)
2,147,730 particles for 13-3 class; 5,987,996 particles for 14-3 class
MiCSPARC seam search pipeline
165,206 particles for 13-3
Volume alignment
Particle recentering
Local refinement
163,421 particles for 13-3
Duplicate removal
Local and CTF refinements
147,409 final particles for 13-3
D
GSFSC Resolution: 3.48Å
No Mask (4.1Å)
Loose (3.7Å)
Tight (3.5Å)
Corrected (3.5Å)
FSC
Spatial frequency-1
E
3072 cFSCs | cFAR: 0.43
mean
[+σ, -σ]
[min, max]
3.95Å
3.19Å
relative frequency
FSC
Spatial frequency-1
F
Elevation
Azimuth
# of images
G
Resolution (Å)

Figure S5 Data processing of the undecorated GDP microtubule reconstruction**. A)** Stick representation views of α/β-tubulin model refined in the undecorated GDP microtubule protofilament density map (transparent grey surface). Map was sharpened using EMReady (He *et al.*, 2023). **B)** Phenix map-model FSC curves for the α/β-tubulin model refined in the undecorated GDP protofilament density map. **C)** Workflow for processing the seam-corrected undecorated 13-3 GDP microtubule using MiCSPARC. **D)** CryoSPARC gold standard FSC curve for the microtubule reconstruction obtained in C). **E)** CryoSPARC cFAR plot for the protofilament reconstruction obtained in C). **F)** CryoSPARC particle orientation distribution for the protofilament reconstruction obtained in C). **G)** Lumenal and side views of the microtubule reconstruction obtained in C) coloured according to local resolution, as calculated in CryoSPARC. The map was postprocessed using EMReady (He et al., 2023).

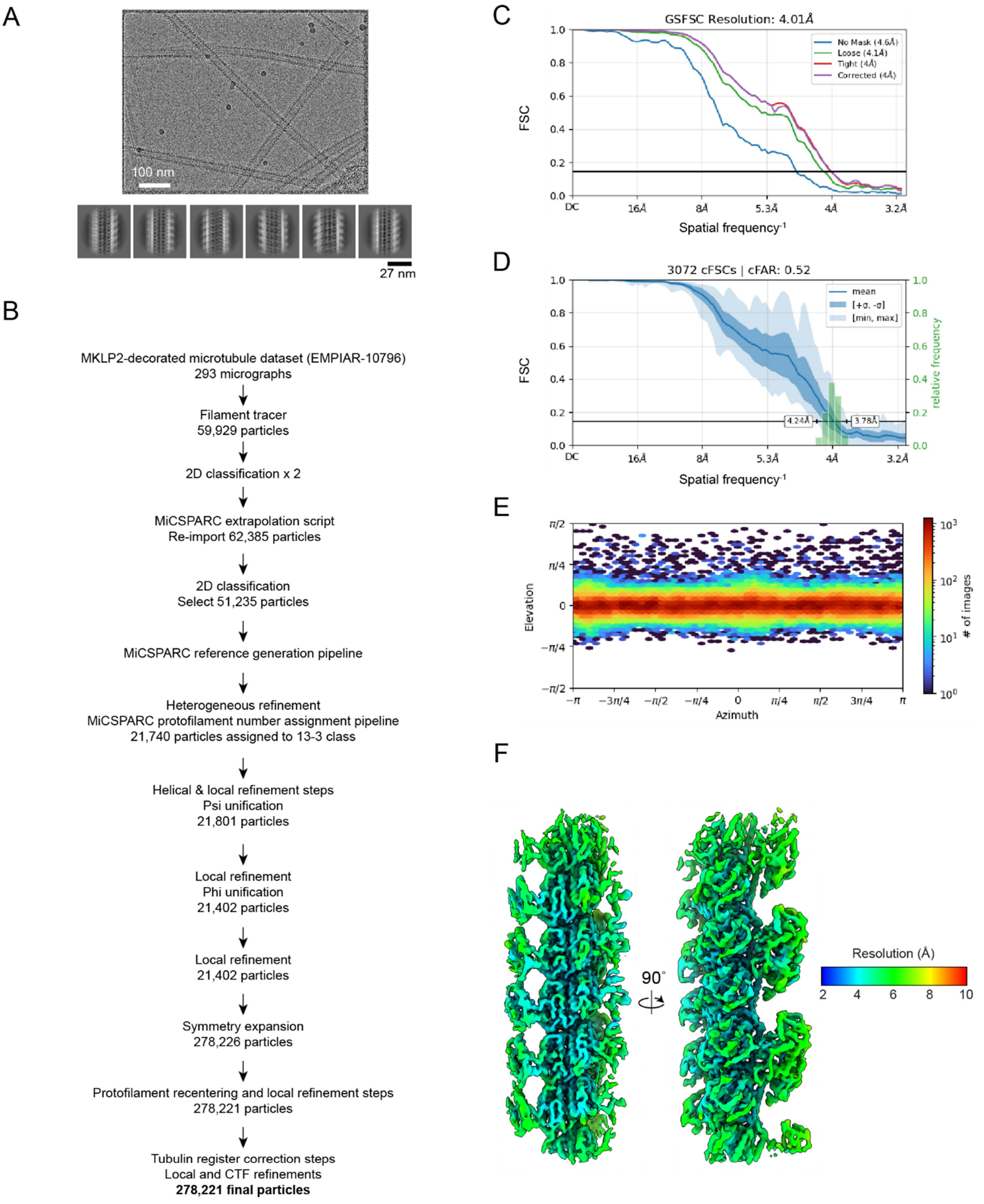
A
100 nm
27 nm
B
MKLP2-decorated microtubule dataset (EMPIAR-10796)
293 micrographs
Filament tracer
59,929 particles
2D classification x 2
MiCSPARC extrapolation script
Re-import 62,385 particles
2D classification
Select 51,235 particles
MiCSPARC reference generation pipeline
Heterogeneous refinement
MiCSPARC protofilament number assignment pipeline
21,740 particles assigned to 13-3 class
Helical & local refinement steps
Psi unification
21,801 particles
Local refinement
Phi unification
21,402 particles
Local refinement
21,402 particles
Symmetry expansion
278,226 particles
Protofilament recentering and local refinement steps
278,221 particles
Tubulin register correction steps
Local and CTF refinements
278,221 final particles
C
GSFSC Resolution: 4.01Å
No Mask (4.6Å)
Loose (4.1Å)
Tight (4Å)
Corrected (4Å)
FSC
Spatial frequency⁻¹
D
3072 cFSCs | cFAR: 0.52
mean
[+σ, -σ]
[min, max]
FSC
relative frequency
4.24Å
3.78Å
Spatial frequency⁻¹
E
Elevation
Azimuth
# of images
F
90°
Resolution (Å)
2
4
6
8
10

Figure S6 Data processing of the MKLP2-decorated protofilament reconstruction**. A)** Top: example cryo-EM micrograph of MKLP2-decorated microtubules, from (Cook *et al.*, 2020). Bottom: example 2D class averages obtained during particle cleanup. **B)** Workflow for processing the register-corrected MKLP2-decorated protofilament reconstruction using MiCSPARC. **C)** CryoSPARC gold standard Fourier shell correlation (FSC) curve for the protofilament reconstruction obtained in B). **D)** CryoSPARC conical FSC Area Ratio (cFAR) plot for the protofilament reconstruction obtained in B). **E)** CryoSPARC particle orientation distribution for the protofilament reconstruction obtained in B). **F)** Lumenal and side views of the protofilament reconstruction obtained in B) coloured according to local resolution, as calculated in CryoSPARC. The map was postprocessed using EMReady (He et al., 2023).

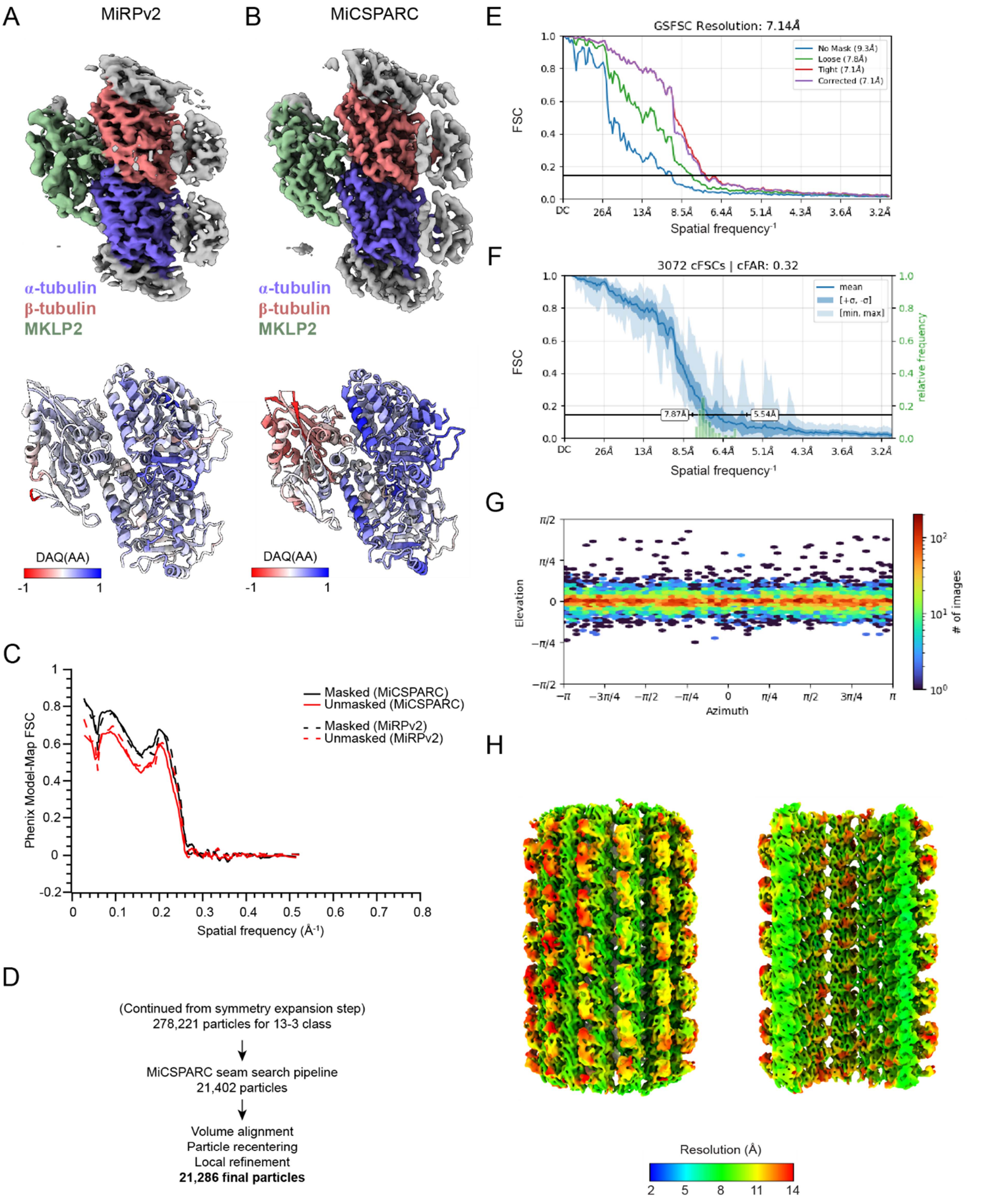
A
MiRPv2
B
MiCSPARC
α-tubulin
β-tubulin
MKLP2
α-tubulin
β-tubulin
MKLP2
DAQ(AA)
-1
1
DAQ(AA)
-1
1
C
Phenix Model-Map FSC
Spatial frequency (Å-1)
Masked (MiCSPARC)
Unmasked (MiCSPARC)
Masked (MiRPv2)
Unmasked (MiRPv2)
D
(Continued from symmetry expansion step)
278,221 particles for 13-3 class
MiCSPARC seam search pipeline
21,402 particles
Volume alignment
Particle recentering
Local refinement
21,286 final particles
E
GSFSC Resolution: 7.14Å
No Mask (9.3Å)
Loose (7.8Å)
Tight (7.1Å)
Corrected (7.1Å)
FSC
Spatial frequency-1
F
3072 cFSCs | cFAR: 0.32
mean
[+σ, -σ]
[min, max]
relative frequency
7.87Å
5.54Å
FSC
Spatial frequency-1
G
Elevation
Azimuth
# of images
H
Resolution (Å)
2
5
8
11
14

Figure S7 Data processing for the MKLP2-decorated microtubule reconstruction. **A)** Top: MiRPv2 reconstruction of MKLP2-decorated tubulin heterodimer, from (Cook *et al.*, 2020). Bottom: refined MKLP2-tubulin model coloured according to DAQ score (Terashi *et al.*, 2022). B) Top: MiCSPARC reconstruction of MKLP2-decorated tubulin heterodimer (this study). Map was sharpened using EMReady (He *et al.*, 2023). Bottom: refined MKLP2-tubulin model coloured according to DAQ score (Terashi *et al.*, 2022). **C)** Phenix map-model FSC curves for the MKLP2-decorated α/β-tubulin models refined in the MiRPv2 (dashed lines) or MiCSPARC (solid lines) protofilament density maps. **D)** Workflow for processing the seam-corrected MKLP2-decorated 13-3 microtubule using MiCSPARC. **E)** CryoSPARC gold standard FSC curve for the microtubule reconstruction obtained in D). **F)** CryoSPARC cFAR plot for the protofilament reconstruction obtained in D). **G)** CryoSPARC particle orientation distribution for the protofilament reconstruction obtained in D). **H)** Lumenal and side views of the microtubule reconstruction obtained in D) coloured according to local resolution, as calculated in CryoSPARC. The map was postprocessed using EMReady (He et al., 2023).

Table S1 Cryo-EM data collection and processing.

	KIF5A-decorated GMPCPP microtubules	*Undecorated GDP microtubules*
Magnification	78,125 X	130,000 X
Voltage (keV)	300	300
Electron exposure (e Å^{-2})	65	80
Defocus range (µm)	-0.3 to -2.9	-0.2 to -3.0
Pixel size (Å)	0.64	1.06
Camera	Gatan K2	Gatan K2
Symmetry imposed	C1 (protofilament and 14-3 microtubule)	C1 (protofilament and 14-3 microtubule)
GIF slit width	20 eV	20 eV
No. initial particle images	55,615	1,440,031
No. final particle images	117,345 (protofilament); 11,523 (14-3 microtubule)	6,499,208 (protofilament); 147,409 (13-3 microtubule)
Map resolution FSC threshold (Å)	2.8 (protofilament); 4.2 (14-3 microtubule	2.9 (protofilament); 3.6 (13-3 microtubule)
Map resolution range (Å)	1.4 to 7.5 (protofilament); 2.8 to 15.0 (14-3 microtubule)	2.4 to 4.8 (protofilament); 2.3 to 8.8 (14-3 microtubule)

Table S2 Model building and refinement.

	KIF5A-decorated GMPCPP microtubules	*Undecorated GDP microtubules*
Initial model used	AlphaFold3	AlphaFold3
Model resolution FSC threshold (Å) (0/0.143/0.5)	2.3/2.5/3.0	2.1/2.5/3.1
Model resolution range (Å)	3.3	2.8
Map sharpening *B* factor (Å^2)	0.0	Not available (LocScale sharpened)
Model composition		
Non-hydrogen atoms	9,327	6,746
Protein/nucleic acid	1173/0	852/1
Ligands	GTP: 1<br>Mg: 2<br>G2P: 1<br>ACP: 1	GTP: 1<br>MG: 1<br>GDP: 1
B factors (Å^2)		
Protein	115.4	50.8
Ligand	121.2	32.5
R.m.s. deviations from ideality		
Bonds (Å)	0.005	0.003
Angles (°)	0.665	0.685
MolProbity score	1.91	2.02
Clashscore	7.19	4.83
Poor rotamers (%)	2.47	3.45
Ramachandran plot		
Favoured (%)	96.65	94.79
Allowed (%)	2.67	4.62
Disallowed (%)	0.69	0.59

Table S3 Cryo-EM data collection and processing for EMPIAR-10796.

	MiCSPARC	*MiRPv2*
Magnification	See EMPIAR-10796	See EMPIAR-10796
Voltage (keV)	See EMPIAR-10796	See EMPIAR-10796
Electron exposure (e Å^{-2})	See EMPIAR-10796	See EMPIAR-10796
Defocus range (µm)	See EMPIAR-10796	See EMPIAR-10796
Pixel size (Å)	See EMPIAR-10796	See EMPIAR-10796
Camera	See EMPIAR-10796	See EMPIAR-10796
Symmetry imposed	C1 (protofilament);<br>C1 (13-3 microtubule)	See EMPIAR-10796
GIF slit width	See EMPIAR-10796	See EMPIAR-10796
No. initial particle images	62,385	See EMPIAR-10796
No. final particle images	278,221 (protofilament);<br>21,286 (13-3 microtubule)	14,411 (See EMD-10131)
Map resolution FSC threshold (Å)	4.0 (protofilament);<br>7.1 (13-3 microtubule)	4.2 (See EMD-10131)
Map resolution range (Å)	3.2 – 10.0 (protofilament);<br>3.2 – 30.0 (13-3 microtubule)	Not available

Table S4 Model building and refinement for EMPIAR-10796.

	MiCSPARC	*MiRPv2*
Initial model used	PDB ID: 5ND4	PDB ID: 5ND4
Model resolution FSC threshold (Å) 0/0.143/0.5	3.4/3.4/4.4	3.7/3.9/4.3
Model resolution range (Å)	4.2	4.1
Map sharpening *B* factor (Å^2)	-203.7	Not available
Model composition		
Non-hydrogen atoms	9,433	9,433
Protein/nucleic acid	1175/1	1175/1
Ligands	ATP: 1<br>ALF: 1<br>GTP: 1<br>TA1: 1	ATP: 1<br>ALF: 1<br>GTP: 1<br>TA1: 1
B factors (Å^2)		
Protein	62.4	83.0
Ligand	65.8	81.9
R.m.s. deviations from ideality		
Bonds (Å)	0.005	0.004
Angles (°)	1.123	0.893
MolProbity score	2.72	2.13
Clashscore	19.52	16.04
Poor rotamers (%)	4.53	0.10
Ramachandran plot		
Favoured (%)	93.29	93.72
Allowed (%)	6.19	5.67
Disallowed (%)	0.52	0.60

S1. Suggested MiCSPARC pipeline

CryoSPARC jobs in bold blue

MiCSPARC jobs in bold orange

MiCSPARC commands highlighted in peach

ChimeraX commands highlighted in grey

PARTICLE PICKING

Preprocessing

1. Run **Patch motion correction**, **Patch CTF estimation**, and **Curate exposures** jobs using default parameters

Generate initial templates for filament tracer

2. Run **Filament tracer**:
    - ~300 Å filament width*, 82 Å separation in diameters (e.g., 0.27 for 300 Å diameter), 300-400 Å template-free diameter
    - If the filament tracer is not picking any microtubules at this stage, increasing the standard deviation (SD) of the Gaussian blur (e.g. to 2.0) and/or increasing both hysteresis values in increments of 1 can help
3. **Inspect picks** if necessary, followed by **Extract from micrographs**:
    - ~550-600 Å box size*, Fourier crop (bin) particles to ~4-5 Å/pixel
    - If tube edges are close to the circular mask (especially with large decorators), expand the box size in later particle extractions
4. Run a **2D Classification** with default parameters
5. **Select 2D** classes with a single clear tube

Generate initial picks

6. **Filament tracer** using the templates selected in step 5
7. **Inspect picks** if necessary, followed by **Extract from micrographs**:
    - ~550-600 Å box size*, Fourier crop (bin) particles to ~4-5 Å/pixel
8. **2D classification**, followed by **Select 2D**:
    - 50-100 classes, depending on the number of particles

- 2 final iterations

9. **2D classification**, followed by **Select 2D**:
   - 50 classes
   - 2 final iterations
   - Disable sigma annealing by setting "Start annealing sigma" at iteration 200
10. **2D classification**:
    - 1 class
    - **Export particles group** when finished

Extrapolate initial picks

The filament tracer will generally not pick along the entire microtubule and may introduce errors in filament grouping. This can be rectified using MiCSPARC's **Filament extrapolation** script:

11. Using the exported directory (it is easiest to run the scripts directly within the exported directory in order to be able to reimport the results group), run:
    ```
    $ python /path/to/csparc_extrapolate_filaments.py --i
    JX_particles_exported.cs --j <number of cpus>
    ```
12. Run **Import result group** using the .csg result file from **Filament extrapolation**: (`/path/to/CS-project/exports/groups/JX_particles/JX_particles_extrapolated.csg`)
13. **Extract from micrographs**
    - Same box size as in step 7
    - Fourier crop (bin) particles to ~2 Å/pixel
14. **2D classification**, followed by **Select 2D**:
    - 50-100 classes, depending on the number of particles
    - 2 final iterations
15. **2D classification**, followed by **Select 2D**:
    - 50 classes
    - 2 final iterations
    - Disable sigma annealing by setting "Start annealing sigma" at iteration 200

PROTOFILAMENT NUMBER SORTING

Generate references

References are required for 3D classification of particles into groups of different protofilament numbers / microtubule lattice architectures. This classification tends to be very dependent on references with the correct factors such as decoration state, positioning, and heterogeneity, and potentially even expansion or compression of the microtubule lattice. Thus, it is highly recommended to create semi-synthetic references directly from the dataset, as follows:

16. Run one **Helical refinement** job for each type of microtubule lattice architecture potentially expected in the dataset
    - Theoretical helical parameters are calculated in the form:

      rise = n_{start} × tubulin spacing ÷ #protofilaments ; and

      twist = (360° + supertwist) ÷ #protofilaments
    - Supertwist can generally be taken as 0 at this stage
    - Common theoretical microtubule parameters (and hence **Helical refinements**):

**Lattice architecture (# pfs - helical "start")**	**Rise (Å)**	**Twist (degrees)**
11-3	11.2	-32.7
12-3	10.3	-30.0
13-3	9.46	-27.7
14-3	8.79	-25.7
15-4	10.9	-24.0
16-4	10.3	-22.5

17. Perform **Symmetry expansion** on the best helical refinement using its refined helical parameters
    - Note: the "best" refinement is not necessarily the one with the highest resolution, but rather the one with the visually best-resolved protofilaments (i.e., no smearing around the tube, ideally distinct tubulin dimers)
18. Recenter the particles on a single protofilament by running **Volume alignment tools** with the "recenter to mask center of mass" option, but first create a mask around a single protofilament:
    - Create a protofilament mask in ChimeraX:
        - First lowpass filter the best helical refinement to 15 Å using **Volume tools**
        - Open map in ChimeraX, set an appropriate masking threshold
        - Tools > Volume Data > Segment Map (may need to change "display at most" parameter to a number much greater than 60)

  - Select a single protofilament
  - File (in the Segment window) > Save selected regions to .mrc file (this is temporary, it doesn't matter where they are saved)
  - In the ChimeraX command line, run:
    `volume resample [new volume id] onGrid #1`
  - Save the resampled volume and import into CryoSPARC using **Import volumes**
  - Lowpass filter the protofilament volume to 15 Å using **Volume tools** (output as mask, and use the threshold chosen above)

19. **Downsample particles** to half of the current box size by cropping in real (not Fourier) space, and recenter particles
20. **Homogeneous reconstruction** of the downsampled particles (mask not required) followed by **Local refinement**:
    - Use volume and particles from **Homogeneous reconstruction** as inputs, but use mask from step 18
    - Use pose/shift gaussian prior during alignment with default values
    - Allow recentering of shifts and rotations after each iteration
21. Use **Volume tools** to inverse the mask from step 18 and crop it in real space to half the box size
22. Perform **Particle subtraction** on the particles from step 20 using the mask from step 21
23. **Homogeneous reconstruction** of the subtracted particles (mask not required) followed by **Local refinement**:
    - Use volume, particles and mask from the **Homogeneous reconstruction** as inputs for the **Local refinement**
    - Use pose/shift gaussian prior during alignment with default values
    - Allow recentering of shifts and rotations after each iteration
    - Enforce non-negativity
    - At the end of the refinement, **Export volume group**
24. Run MiCSPARC's **Reference generation** script:
    ```
    $ python /path/to/csparc_create_pfn_references.py --i
    JX_volume_exported.cs ...
    ```
    The `--recenter` coordinates are those reported by CryoSPARC in the volume alignment step 18 ("New center will be located at voxel coordinates:"). These coordinates must be input here in the format `--recenter "x, y, z"`

Protofilament number assignment

25. Import the various reference volumes into CryoSPARC using **Import 3D volumes**
26. Run **Heterogeneous refinement** on the particles from step 15 using these imported volumes as references
    - Force hard classification
    - If any of the output classes have 0 particles assigned to it, remove the corresponding reference and re-run the job
    - **Export particles group** when finished
27. Run MiCSPARC's **Protofilament number assignment** script:
    ```
    $ python /path/to/csparc_assign_pfns.py --i ...
    ```
28. **Import result group** using the .csg result file from **Protofilament number assignment**
29. Run **Heterogeneous reconstruction** on the imported particles
    - Note: an error will occur if there are any classes with zero particles from the step 26 heterogeneous refinement; in this case re-run the classification without that reference and repeat steps 27-29.
30. Run a **Split volume groups** job on the resulting particles

ROUGH MICROTUBULE ALIGNMENT

Having each particle along the microtubule in the same orientation is important for later symmetry expansion and seam searching steps, as it allows us to assign each group of expansions as the same protofilament along the microtubule. For each good 3D class from steps 29-30 (or you can focus on just one dominant class, the number of particles is often enough):

31. Run a **Helical refinement** with the corresponding theoretical helical parameters
    - Minimise per particle scale
    - Use non-uniform refinement
    - Note: particles may need to be unbinned and re-extracted if they hit Nyquist
    - Note: if the "good" 3D class(es) contain a particle number greater than 50-100k, computational resources may be a problem due to the large box sizes required for unbinned microtubule segments combined with downstream symmetry expansion operations. In this case, it is probably best to repeat steps 1-30 with a randomized subset of micrographs (not particles, since particles must remain linked to filaments and micrographs in order for seam correction etc. to work).
32. Run a **Local CTF refinement** followed by a **Global CTF refinement**

- Use the particles, volume and mask from the **Helical refinement** as an input to the **Local CTF refinement**; then do the same for the **Global CTF refinement**, just update the particles to come from the **Local CTF refinement** job

33. Run a **Helical refinement** on the CTF-refined particles
    - Do not impose helical symmetry (leave helical rise & twist fields blank)
    - Minimise per-particle scale
    - Use non-uniform refinement
    - **Export particles group** when finished
34. Run MiCSPARC's **Psi unification** script:

```
$ python /path/to/csparc_unify_psi.py ...
```

35. **Import result group** using the .csg result file from **Psi unification**
36. Run a **Local refinement**
    - Use the resulting helical volume and mask from step 33
    - Use pose/shift gaussian prior during alignment with 5 SD for rotations and 3 SD for shifts
    - Allow rotation recentering
    - **Export particles group** when finished
37. Run MiCSPARC's **Phi unification** script:

```
$ python /path/to/csparc_unify_phi.py ...
```

38. **Import result group** using the .csg result file from **Phi unification**
39. Run a **Local refinement**
    - Again use the resulting helical volume and mask from step 33
    - Use pose/shift gaussian prior during alignment with 5 SD for rotations and 3 SD for shifts
    - Allow rotation recentering
    - (Optional) Force re-do GS split
    - **Export particles group** when finished
40. Run MiCSPARC's **Phi unification** script again:

```
$ python /path/to/csparc_unify_phi.py ...
```

41. **Import result group** using the .csg result file from the second **Phi unification**
42. Run a **Local refinement**
    - Again use the resulting helical volume and mask from step 33
    - Use pose/shift gaussian prior during alignment with 3 SD for rotations and 2 SD for shifts
    - Do not allow any recentering
43. Perform **Symmetry expansion** on the refined particles
    - Use refined helical parameters from step 31 with order = protofilament #

    - Alternatively, run a **Symmetry search** job on output of step 42, and use the resulting parameters

44. Run a **Local refinement**
    - Again use the resulting helical volume and mask from step 33
    - Use pose/shift gaussian prior during alignment with 3 SD for rotations and 2 SD for shifts
    - Do not allow any recentering
    - Note: for undecorated microtubules, if this step does not reach <4 Å resolution, later steps may prove difficult or even impossible. Decorated particles will be more lenient depending on the size of the decorator.

PROTOFILAMENT ALIGNMENT

45. Create a mask around a single protofilament in the resulting volume from step 44 using ChimeraX, as in step 18
    - Note: if refining multiple microtubule lattice arrangements, it can be helpful to select the protofilament that coincides best across all microtubule types.
    - Note: with large decorators, select the protofilament with the most mixed population of decorator registers to ensure no gaps in the mask. Selecting the “worst” protofilament will also give the best split for the register classification, which will generally allow seam search to work better.
    - Note: a tight mask along the protofilament and including the width of a single tubulin dimer plus the decorator can be beneficial at this stage; several masks may need to be tested to optimize the pipeline for different decorators.
46. Recenter the particles from step 44 on this protofilament by running **Volume alignment tools** with the “recenter to mask center of mass” option selected
47. Use **Volume tools** to crop the new realigned mask to half the original box size and invert
    - Note: for this as well as the local refinement mask in step 49 below, it may be best to first re-run **Volume alignment tools** on the imported volume from step 45 and generate cropped masks on the centered volume. Generating masks from masks may not correctly propagate soft edges, thresholds etc.
48. **Extract from micrographs**
    - Same box size as in step 47
    - Recenter using all aligned shifts

49. **Homogeneous reconstruction** of the extracted particles (mask not required) followed by **Local refinement**:
    - Use volume and particles from the **Homogeneous reconstruction** as inputs, but run **Volume tools** to generate and use a new, non-inverted mask from step 47
    - Use pose/shift gaussian prior during alignment with 3 SD for rotations and 2 SD for shifts
    - Do not allow any recentering
50. Perform **Particle subtraction** on the particles from step 49 using the mask from step 47
51. **Homogeneous reconstruction** of the subtracted particles (mask not required) followed by **Local refinement**:
    - Use volume and particles from the **Homogeneous reconstruction** as inputs and use the non-inverted mask from step 49
    - Use pose/shift gaussian prior during alignment with 3 SD for rotations and 2 SD for shifts
    - Allow recentering of shifts and rotations after each iteration
    - Enforce non-negativity

If refining multiple classes:

52. Pick a base refinement, align other protofilament number reconstructions with **Align 3D maps** (may need to be roughly aligned manually with volume alignment and ChimeraX first)
53. **Homogeneous reconstruction** of all combined particles (mask not required) followed by **Local refinement**:
    - Use volume and particles from the **Homogeneous reconstruction** as inputs and use the non-inverted mask from step 49
    - Use pose/shift gaussian prior during alignment with 3 SD for rotations and 2 SD for shifts
    - Allow recentering of shifts and rotations after each iteration
    - Enforce non-negativity

Protofilament register correction

54. Perform **3D classification** of the refined particles from step 53:
    - 10 classes
    - Filter resolution to 4 Å for undecorated microtubules and up to 12 Å for large decorators (lower resolution limits tends to allow the classification to work better for decorators)
    - Per-particle scale = none
55. Identify "good" reference volumes:
    - For undecorated microtubules: at least one class with a distinct α-tubulin S9-S10 loop, ideally at least two which exhibit a difference in register

   - For decorated microtubules: at least one class with clear and convincing spacing of decorator, ideally at least two which exhibit a difference in register (if applicable)
56. For each “good” volume, perform **Homogeneous reconstruction** (mask not required) followed by **Local refinement**:
   - Use volume and particles from the **Homogeneous reconstruction** as inputs and use the non-inverted mask from step 49
   - Use pose/shift gaussian prior during alignment with 3 SD for rotations and 2 SD for shifts
   - Allow recentering of shifts and rotations after each iteration
   - Enforce non-negativity
57. If only one register was observed, create the other register using **Volume alignment tools**:
   - Shift in Z by ~41 Å
58. Perform **3D classification** of the refined particles from step 53
   - 2 classes (use the refined volumes in step 56 as reference volumes)
   - Filter resolution to 4 Å for undecorated microtubules and up to 12 Å for large decorators
   - Input initialization mode
   - Per-particle scale = none
   - Note: for undecorated datasets, if everything is aligned well so far, this step should result in a ~50/50 split of particles. The distribution of decorated datasets may depend on how well-resolved the seam was prior to symmetry expansion (step 43).
59. For each class, perform **Homogeneous reconstruction** (mask not required) followed by **Local refinement**:
   - Use volume, particles and mask from the **Homogeneous reconstruction** as inputs
   - Use pose/shift gaussian prior during alignment with 3 SD for rotations and 2 SD for shifts
   - Allow recentering of shifts and rotations after each iteration
   - Enforce non-negativity
60. Use **Volume alignment tools** to shift the lower-resolution class in Z by ~41 Å
61. Use **Align 3D maps** to align the shifted class (maps & particles) to the unshifted, higher-resolution class
   - Update particle alignments
62. Run **Homogeneous reconstruction** (mask not required) followed by **Local refinement**:
   - Use both shifted and unshifted particles (steps 59 and 61)
   - Use volume, particles and mask from the **Homogeneous reconstruction** as inputs
   - Use pose/shift gaussian prior during alignment with 3 SD for rotations and 2 SD for shifts
   - Allow recentering of shifts and rotations after each iteration

- Enforce non-negativity

63. Run a **Local CTF refinement** followed by a **Global CTF refinement**
    - Use the particles, volume and mask from step 62 as inputs to the **Local CTF refinement**; then do the same for the **Global CTF refinement**, just update the particles to come from the **Local CTF refinement** job
64. Run a **Local refinement**:
    - Use volume and mask from the **Local refinement** in step 62, and particles from the **Global CTF refinement** in step 63, as inputs
    - Use pose/shift gaussian prior during alignment with 3 SD for rotations and 2 SD for shifts
    - Allow recentering of shifts and rotations after each iteration
    - Enforce non-negativity

To further improve resolution of protofilament reconstruction:

65. Remove duplicate particles in the final refinement from step 64 using **Duplicate removal** with default settings
66. Perform **Reference-based motion refinement** on these particles using default settings
67. Run a **Local refinement**:
    - Use volume, particles and mask from the **Local refinement** in step 62 as inputs
    - Use pose/shift gaussian prior during alignment with 3 SD for rotations and 2 SD for shifts
    - Allow recentering of shifts and rotations after each iteration
    - Enforce non-negativity
    - Sharpen the map either with **Sharpening tools** or with a deep learning method such as EMReady

SEAM-CORRECTED MICROTUBULE RECONSTRUCTION

68. Perform a **Heterogeneous reconstruction** using pre-duplicate removal particles (step 64)
    - Be sure to link the `alignments3D_multi` field of the 2-class 3D classification in step 58
    - **Export particles group** when finished
69. Run MiCSPARC's **Seam search** script:

```
$ python /path/to/csparc_seam_search.py …
```

    - Use the recenter coords from step 46, taking account for differences in the pixel size between step 46 and step 48 in case they were changed

70. **Import result group** using the .csg result file from the **Seam search** step (or groups, if analyzing multiple protofilament number microtubules; (e.g. `/path/to/CS-project/exports/groups/JX_particles/JX_particles_seamed_50_13pf.csg`)
71. Recenter the particles from step 70 on the coordinates output by the **Seam search** script using **Volume alignment tools**
72. **Extract from micrographs**
    - Return to original microtubule filament box size
    - Recenter using all aligned shifts
73. Run **Homogeneous reconstruction** (mask not required) followed by **Local refinement**:
    - Use volume and mask from step 33, and particles from the **Homogeneous reconstruction** as inputs
    - Use pose/shift gaussian prior during alignment with 3 SD for rotations and 2 SD for shifts
    - Don't allow any recentering
74. Run a **Local CTF refinement** followed by a **Global CTF refinement**
    - Use the particles, volume and mask from step 73 as inputs to the **Local CTF refinement**; then do the same for the **Global CTF refinement**, just update the particles to come from the **Local CTF refinement** job
75. Run a **Local refinement**:
    - Use volume and mask from the **Local refinement** in step 73, and particles from **Global CTF refinement** in step 74, as inputs
    - Use pose/shift gaussian prior during alignment with 3 SD for rotations and 2 SD for shifts
    - Don't allow any recentering

Not strictly necessary, but to potentially improve the signal of decorators and/or the quality of the final microtubule reconstruction, one can try to:

76. Remove duplicate particles in the final refinement from step 75 using **Duplicate removal** with default settings
77. Perform a **3D Classification** job:
    - 4-10 classes
    - Filter resolution to 4 Å
    - Force hard classification
78. Perform **Reference-based motion refinement** on particles from the best class using default settings
79. Run a **Local refinement** on these final particles:

- Use volume and mask from the **Local refinement** in step 75, and particles from step 78 as inputs
- Use pose/shift gaussian prior during alignment with 3 SD for rotations and 2 SD for shifts
- Don't allow any recentering

80. Sharpen the map either with **Sharpening tools** or with a deep learning method such as EMReady

S2. MiCSPARC automation

If suitable 3D references for 11 to 16-protofilament microtubules exist, the first half of the pipeline (Steps 1-15 and 25-44, Appendix 1) requires no human intervention, and has been automated via cryosparc-tools and tested against CryoSPARC version 4.7.

The automation pipeline requires the user to set up a configuration file that will allow the software to connect directly to the user's CryoSPARC instance, import movies into CryoSPARC, and specify several variables related to imported movies to process, particle extraction and picking. The automation should be run from a persistent terminal that has access to the CryoSPARC project directories, e.g. a 'tmux' or 'screen' session on the CryoSPARC or HPC server. Examples of the required configuration files are provided in the MiCSPARC GitHub repository.

The automation outputs a set of angle-unified, symmetry-expanded microtubule reconstructions that contain enough particles for protofilament alignment to high resolution (Step 45, Appendix 1).

MiCSPARC automation

Configuration of the instance

1. Create a 'cs_config.yml' file in the user's home directory, defined by the terminal's $HOME variable that describes the CryoSPARC instance as shown in the 'csparc_automation/cs_config.yml' example file in the GitHub repository.
2. The variables defined here allow connection directly to the CryoSPARC server and specify several lanes to dispatch jobs to. Lanes can all be the same, e.g. 'default' on a single master-worker workstation. The location for 11-16 protofilament microtubule models is also specified here.
3. The specified microtubule models search path should match exactly 6 volumes, that can be alphabetically sorted from 11 to 16 protofilaments. Name starting with number of protofilaments is recommended.
4. Link MiCSPARC scripts into csparc_automation directory:
   ```
   $ cd /path/to/MiCSPARC/csparc_automation/
   $ ln -s ../csparc_*.py .
   ```

Automated processing

1. Import movies into CryoSPARC
2. Run the automation pipeline:
```
$ python /path/to/MiCSPARC/csparc_automation/cs_autopick.py
```
3. The automation creates a logfile, 'autopick.log' in the CryoSPARC project directory. If the processing crashes, it can be restarted by running the automation pipeline from this directory. It will read the present logfile, rerun the last step that CryoSPARC logs as 'finished', or the first step that is not logged as 'finished', and continue from there.
4. When finished, continue the MiCSPARC pipeline from protofilament alignment (Step 45, Appendix 1)

Caveats

1. The success rate of the automated processing pipeline heavily depends on the quality of the data and of the microtubule references.
2. With poor or sparse data, automated particle sorting may fail. In these cases, manual selection of classes can be employed, and the pipeline restarted from the next step by manually editing the autopick.log file.
3. If heterogeneous refinement (protofilament sorting) fails to produce good reconstructions, creation of new references from the data directly (Step 16-24, Appendix 1) is recommended.
4. If heterogeneous reconstruction fails, this is due to one or more classes assigned zero particles in the preceding heterogeneous refinement. In this case, rerun the heterogeneous refinement omitting the respective references, and continue with the standard MiCSPARC pipeline manually (Step 26, Appendix 1).

References

Akhmanova, A., and M.O. Steinmetz. 2015. Control of microtubule organization and dynamics: two ends in the limelight. *Nat. Rev. Mol. Cell Biol.* 16:711–726.

Alushin, G.M., G.C. Lander, E.H. Kellogg, R. Zhang, D. Baker, and E. Nogales. 2014. High-resolution microtubule structures reveal the structural transitions in αβ-tubulin upon GTP hydrolysis. *Cell*. 157:1117–1129.

Atherton, J., I.-M. Yu, A. Cook, J.M. Muretta, A. Joseph, J. Major, Y. Sourigues, J. Clause, M. Topf, S.S. Rosenfeld, A. Houdusse, and C.A. Moores. 2017. The divergent mitotic kinesin MKLP2 exhibits atypical structure and mechanochemistry. *Elife*. 6. doi:10.7554/eLife.27793.

Brouhard, G.J., and L.M. Rice. 2014. The contribution of αβ-tubulin curvature to microtubule dynamics. *J. Cell Biol.* 207:323–334.

Brouhard, G.J., and L.M. Rice. 2018. Microtubule dynamics: an interplay of biochemistry and mechanics. *Nat. Rev. Mol. Cell Biol.* 19:451–463.

Castoldi, M., and A.V. Popov. 2003. Purification of brain tubulin through two cycles of polymerization-depolymerization in a high-molarity buffer. *Protein Expr. Purif.* 32:83–88.

Cook, A.D., S.W. Manka, S. Wang, C.A. Moores, and J. Atherton. 2020. A microtubule RELION-based pipeline for cryo-EM image processing. *J. Struct. Biol.* 209:107402.

Debs, G.E., M. Cha, X. Liu, A.R. Huehn, and C.V. Sindelar. 2020. Dynamic and asymmetric fluctuations in the microtubule wall captured by high-resolution cryoelectron microscopy. *Proc. Natl. Acad. Sci. U. S. A.* 117:16976–16984.

Emsley, P., B. Lohkamp, W.G. Scott, and K. Cowtan. 2010. Features and development of Coot. *Acta Crystallogr. D Biol. Crystallogr.* 66:486–501.

He, J., T. Li, and S.-Y. Huang. 2023. Improvement of cryo-EM maps by simultaneous local and non-local deep learning. *Nat. Commun.* 14:3217.

Hyman, A.A., S. Salser, D.N. Drechsel, N. Unwin, and T.J. Mitchison. 1992. Role of GTP hydrolysis in microtubule dynamics: information from a slowly hydrolyzable analogue, GMPCPP. *Mol. Biol. Cell*. 3:1155–1167.

Lacey, S.E., S. He, S.H. Scheres, and A.P. Carter. 2019. Cryo-EM of dynein microtubule-binding domains shows how an axonemal dynein distorts the microtubule. *Elife*. 8. doi:10.7554/eLife.47145.

Menéndez, M., G. Rivas, J.F. Díaz, and J.M. Andreu. 1998. Control of the structural stability of the tubulin dimer by one high affinity bound magnesium ion at nucleotide N-site. *J. Biol. Chem.* 273:167–176.

Nogales, E., S. Wolf, I.A. Khan, R. Luduena, and K. Downing. 1995. Structure of tubulin at 6.5 Å and location of the taxol-binding site. *Nature*. 375:424–427.

Olieric, N., M. Kuchen, S. Wagen, M. Sauter, S. Crone, S. Edmondson, D. Frey, C. Ostermeier, M.O. Steinmetz, and R. Jaussi. 2010. Automated seamless DNA co-transformation cloning with direct

expression vectors applying positive or negative insert selection. *BMC Biotechnol.* 10:56.

Pettersen, E.F., T.D. Goddard, C.C. Huang, E.C. Meng, G.S. Couch, T.I. Croll, J.H. Morris, and T.E. Ferrin. 2021. UCSF ChimeraX: Structure visualization for researchers, educators, and developers. *Protein Sci.* 30:70–82.

Punjani, A., J.L. Rubinstein, D.J. Fleet, and M.A. Brubaker. 2017. cryoSPARC: algorithms for rapid unsupervised cryo-EM structure determination. *Nat. Methods*. 14:290–296.

Scheres, S.H.W. 2012. RELION: Implementation of a Bayesian approach to cryo-EM structure determination. *J. Struct. Biol.* 180:519–530.

Sui, H., and K.H. Downing. 2010. Structural basis of interprotofilament interaction and lateral deformation of microtubules. *Structure*. 18:1022–1031.

Terashi, G., X. Wang, S.R. Maddhuri Venkata Subramaniya, J.J.G. Tesmer, and D. Kihara. 2022. Residue-wise local quality estimation for protein models from cryo-EM maps. *Nat. Methods*. 19:1116–1125.

Ti, S.-C., G.M. Alushin, and T.M. Kapoor. 2018. Human β-Tubulin Isotypes Can Regulate Microtubule Protofilament Number and Stability. *Dev. Cell*. 47:175-190.e5.

Wade, R.H., D. Chrétien, and D. Job. 1990. Characterization of microtubule protofilament numbers. How does the surface lattice accommodate? *J. Mol. Biol.* 212:775–786.

Zhang, R., G.M. Alushin, A. Brown, and E. Nogales. 2015. Mechanistic Origin of Microtubule Dynamic Instability and Its Modulation by EB Proteins. *Cell*. 162:849–859.

Zhang, R., B. LaFrance, and E. Nogales. 2018. Separating the effects of nucleotide and EB binding on microtubule structure. *Proc. Natl. Acad. Sci. U. S. A.* 115:E6191–E6200.

Zhang, R., and E. Nogales. 2015. A new protocol to accurately determine microtubule lattice seam location. *J. Struct. Biol.* 192:245–254.
